# Reduction of *Drosophila* Mitochondrial RNase P in Skeletal and Heart Muscle Causes Muscle Degeneration, Cardiomyopathy, and Heart Arrhythmia

**DOI:** 10.3389/fcell.2022.788516

**Published:** 2022-05-19

**Authors:** Maithili Saoji, Courtney E. Petersen, Aditya Sen, Benjamin A. Tripoli, Jeremy T. Smyth, Rachel T. Cox

**Affiliations:** ^1^ Department of Biochemistry and Molecular Biology, Uniformed Services University, Bethesda, MD, United States; ^2^ Henry M. Jackson Foundation, Bethesda, MD, United States.; ^3^ Department of Anatomy, Physiology and Genetics, Uniformed Services University, Bethesda, MD, United States

**Keywords:** mitochondrial RNase P, mitochondrial disease, cardiomyopathy, arrythmia, skeletal muscle, MRPP, drosophila, intravital imaging

## Abstract

In this study, we examine the cause and progression of mitochondrial diseases linked to the loss of mtRNase P, a three-protein complex responsible for processing and cleaving mitochondrial transfer RNAs (tRNA) from their nascent transcripts. When mtRNase P function is missing, mature mitochondrial tRNA levels are decreased, resulting in mitochondrial dysfunction. mtRNase P is composed of Mitochondrial RNase P Protein (MRPP) 1, 2, and 3. MRPP1 and 2 have their own enzymatic activity separate from MRPP3, which is the endonuclease responsible for cleaving tRNA. Human mutations in all subunits cause mitochondrial disease. The loss of mitochondrial function can cause devastating, often multisystemic failures. When mitochondria do not provide enough energy and metabolites, the result can be skeletal muscle weakness, cardiomyopathy, and heart arrhythmias. These symptoms are complex and often difficult to interpret, making disease models useful for diagnosing disease onset and progression. Previously, we identified *Drosophila* orthologs of each mtRNase P subunit (Roswell/MRPP1, Scully/MRPP2, Mulder/MRPP3) and found that the loss of each subunit causes lethality and decreased mitochondrial tRNA processing *in vivo*. Here, we use *Drosophila* to model mtRNase P mitochondrial diseases by reducing the level of each subunit in skeletal and heart muscle using tissue-specific RNAi knockdown. We find that mtRNase P reduction in skeletal muscle decreases adult eclosion and causes reduced muscle mass and function. Adult flies exhibit significant age-progressive locomotor defects. Cardiac-specific mtRNase P knockdowns reduce fly lifespan for Roswell and Scully, but not Mulder. Using intravital imaging, we find that adult hearts have impaired contractility and exhibit substantial arrhythmia. This occurs for *roswell* and *mulder* knockdowns, but with little effect for *scully*. The phenotypes shown here are similar to those exhibited by patients with mitochondrial disease, including disease caused by mutations in MRPP1 and 2. These findings also suggest that skeletal and cardiac deficiencies induced by mtRNase P loss are differentially affected by the three subunits. These differences could have implications for disease progression in skeletal and heart muscle and shed light on how the enzyme complex functions in different tissues.

## Introduction

Mitochondrial function is a critical metabolic nexus for ATP production, fatty acid beta-oxidation, and important intermediate metabolites. The proteins required for these processes are encoded in the nucleus and mitochondrial DNA (mtDNA). The latter encodes for a small number of the thousands of proteins required for mitochondrial function. Spontaneous mtDNA mutations can arise during embryogenesis, causing mitochondrial disease in children and young adults. mtDNA mutations often accumulate in the maternal germline and are passed from mother to child. Since the majority of proteins required for mitochondrial function are encoded in the nucleus, inherited chromosomal mutations may also cause mitochondrial disease (Craven et al., 2017).

Symptoms of mitochondrial disease are often manifested in organs and tissues with high energy needs, reflecting the fundamental cellular role of mitochondria in the generation of ATP. Patients often suffer from myopathies that affect smooth, skeletal, and cardiac muscles (Pfeffer and Chinnery, 2013), with defects in smooth muscle leading to gastrointestinal problems, including trouble swallowing, constipation, and diarrhea; defects in the skeletal muscle causing weakness, exercise intolerance, and hypotonia; and finally cardiomyopathies, particularly in children (El-Hattab and Scaglia, 2016). Because they are multisystemic, mitochondrial diseases are challenging to treat with more than palliative care. Disease models of mitochondrial disease offer the opportunity to not only learn more about the basic biology underpinning disease symptoms but also to test potential treatments [reviewed in (Ruzzenente et al., 2016; Sen and Cox, 2017)].

**FIGURE 1:**
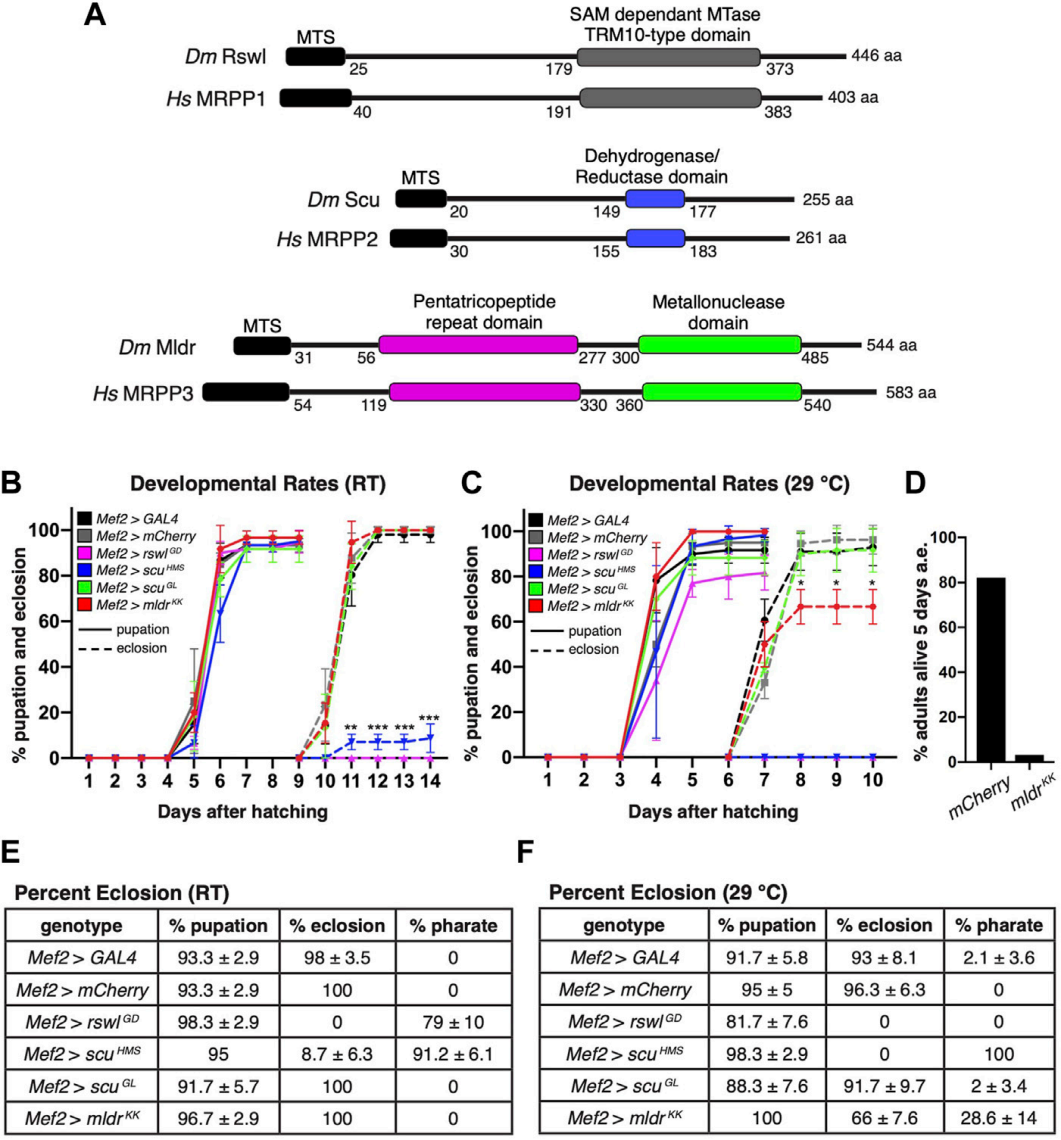
Reduction of mtRNase P subunits in skeletal muscle causes pupation and eclosion defects. **(A)** Schematic of conserved domains of mtRNase P for *Drosophila* and humans. MTS = mitochondrial targeting sequence MTase = methyltransferase. **(B,C)** Pupation (solid lines) and eclosion (dashed lines) rates at room temperature (RT) **(B)** and 29°C **(C)** for larvae expressing mtRNase P RNAi using the muscle-specific *Mef2* GAL4 driver. **(D)** Percent *Mef2* > *mldr* ^*KK*^ flies alive 5 days post-eclosion at 29°C. Numbers are the aggregate of viable adults from three vials after 5 days and have no error bar. a.e. = after eclosion. **(E,F)** Percent pupation, eclosion, and pharate adults at room temperature (RT) and 29°C after expressing *mtRNase P* RNAi using *Mef2* GAL4 to drive expression. *GAL4* RNAi and *mCherry* RNAi serve as controls. **(B,C)** Error bars = s.e.m. Statistical significance was calculated with GraphPad Prism using one unpaired t-test per time point between *GAL4* control RNAi and experimental RNAi to calculate individual *p* values. Only time points marked with an asterisk show a difference. All overtime points were not statistically different from controls. * = *p* < 0.02, ** = *p* < 0.001, *** = *p* < 0.0001. **(E,F)** ± standard deviation. Graphs were plotted using GraphPad Prism.

In this study, we examine mitochondrial RNase P (mtRNase P), an enzyme complex essential for mitochondrial function. mtRNase P is a three-protein complex required to cleave the 5′- end of mt:tRNAs (Holzmann et al., 2008). It is comprised of mitochondrial RNase P Protein 1 (MRPP1), MRPP2, and MRPP3 (Figure 1A) (Holzmann et al., 2008). While each protein has a distinct stand-alone enzymatic function, all three proteins function together to process mt:tRNAs. MRPP1 (gene = *TRMT10C*) is a methyltransferase; MRPP2 (gene = *HSD17B10*) is a dehydrogenase; and MRPP3 (gene = *PRORP*) is an endonuclease. MRPP1 and 2 form a subcomplex that binds to nascent mt:tRNA and recruits MRPP3, while MRPP3 is responsible for cleaving the tRNA (Holzmann et al., 2008; Vilardo et al., 2012; Deutschmann et al., 2014). Presently, mitochondrial diseases have been associated with mutations in all three subunits, although with variable clinical phenotypes [reviewed in (Saoji and Cox, 2018)]. Mitochondrial disease associated with mutations in MRPP2, called HSD10 disease, is multisystemic causing cardiomyopathy, hypotonia, and neurodegeneration (Zschocke, 2012) in which the most severe forms affect infants. Two identified mutations in MRPP1 caused infant death, with both children having hypotonia and one with ventricular hypertrophy (Metodiev et al., 2016). Finally, mtDNA mutations at the mtRNase P cleavage site have been shown to cause severe maternally-inherited hypertension and other cardiac problems, and mutations in MRPP3 cause hypotonia, learning disabilities, and deafness (Hochberg et al., 2021; Wang et al., 2011; Zhu et al., 2009).

With respect to mtRNase P, *Drosophila* contains single MRPP homologs: Roswell (Rswl)/MRPP1, Scully (Scu)/MRPP2, and Mulder (Mldr)/MRPP3 (Figure 1A), all of which are pupal lethal when mutated (Sen et al., 2016; Saoji et al., 2021). *rswl*, *scu,* and *mldr* mutants have decreased ATP levels, disrupted mitochondrial morphology, and abnormal accumulation of large mtRNA species (Sen et al., 2016; Saoji et al., 2021). In addition, mt:tRNA cleavage at specific junctions in different mtRNA polycistronic transcripts is differentially affected by the loss of individual subunits of mtRNase P (Saoji et al., 2021). For example, loss of Rswl is most disruptive to mt:tRNA processing while loss of Scu sometimes has no effect even though the mtRNase P complex consists of three proteins.

This study focuses on how the loss of mtRNase P adversely affects mitochondrial function and the progression of mitochondrial diseases. To do this, we used tissue-specific RNAi knockdown of each subunit to study the effect of reduction of Rswl, Scu, and Mldr on the skeletal and cardiac muscle. We find that reduced mtRNase P in skeletal muscle does not affect pupation but does disrupt eclosion. In addition, muscle size is reduced and wing posture abnormal. Finally, muscle knockdown of *scu* and *mldr* leads to age-progressive loss of locomotion. Heart-specific knockdown affects neither pupation nor eclosion but reduces lifespan for flies lacking *rswl* and *scu*. Using intravital imaging, we find contractility is impaired and rhythmicity is greatly reduced in flies expressing heart-specific *rswl* and *mldr* RNAi, but not *scu*. These skeletal and cardiac phenotypes recapitulate symptoms in patients with mitochondrial diseases. Furthermore, cardiac-specific knockdown revealed tissue-specific effects of Mldr, suggesting tissues may be differentially affected by the loss of mtRNase P subunits.

## Materials and Methods

### Fly Stocks

Y w was used as the wild type control. The following stocks were obtained from the Bloomington *Drosophila* Stock Center, Bloomington, IN, United States.: y[1] v[1]; P{y[+t7.7] v[+t1.8] = TRiP.HMS02305}attP40 (BDSC Cat# 41884, RRID:BDSC_41884), y[1] sc[*] v[1] sev[21]; P{y[+t7.7] v[+t1.8] = TRiP.GL01079}attP2 (BDSC Cat# 42476, RRID:BDSC_42476), y[1] sc[*] v[1] sev[21]; P{y[+t7.7] v[+t1.8] = VALIUM20-GAL4.2}attP2 (BDSC Cat# 35783, RRID:BDSC_35783), y[1] sc[*] v[1] sev[21]; P{y[+t7.7] v[+t1.8] = VALIUM20-mCherry}attP2 (BDSC Cat# 35785, RRID:BDSC_35785), y[1] w[*]; P{w[+mC] = GAL4-Mef2.R}3 (BDSC Cat# 27390, RRID:BDSC_27390). The following stocks were obtained from the Vienna *Drosophila* Resource Center, Vienna, Austria: rswl[GD12447] (FlyBase Cat# FBst0457384, RRID:FlyBase_FBst0457384) and y w[1118]/mldr[KK108043] (FlyBase Cat# FBst0478467, RRID:FlyBase_FBst0478467). CM-tdTomato flies were a gift from Dr. Rolf Bodmer (Sanford Burnham Prebys Medical Discovery Institute, La Jolla, CA, United States). 4xHand-GAL4 was a gift from Dr. Zhe Han (Zhu et al., 2017) (University of Maryland School of Medicine, Baltimore, MD, United States).

### Pupation, Eclosion, Survival, and Wing Analysis

Twenty-first instar larvae collected 1 day after egg laying were transferred into fly food vials and grown at room temperature or 29°C. The number of pupae was counted every 24 h after the onset of pupation. The number of eclosed adults was counted each day after the onset of eclosion. For each genotype, the experiment was performed in triplicate. For adult survival experiments, 20 adult males or females were placed in vials and kept at room temperature or 29°C. Virgin females were collected and allowed to mate for 24 h after which the females were separated from males and transferred to vials. The number of surviving adults was counted every 24 h and the flies were transferred to fresh food every 2 to 3 days. For each genotype, the experiment was performed in triplicate. The average and standard deviation calculations per timepoint for pupation and eclosion were done using GraphPad Prism and Microsoft Excel. The significance between the survival distributions was calculated using Online Application for Survival Analysis 2 (OASIS 2) (Han et al., 2016) and Log-Rank analysis. Curve shapes were created using Kolmogorov–Smirnov Test. The survival graphs showing average survival with error bars were plotted using GraphPad Prism. For wing analysis, adult wings were removed from the fly with a small surgical scissors, and rinsed with antibody wash (1x PBS, 0.1% Triton X-100x, 0.1% bovine albumin) then mounted in 50% glycerol. Pictures were taken using Accu-scope 3076 digital microscope 0.67x–4.5x with AU-600-HDS (Excelis camera).

### Negative Geotaxis

0–24-hour females or males of each genotype were collected and kept at either room temperature or 29°C. At 7-days, 20 flies were transferred to an empty vial with an 8 cm marking. The flies were tapped down and allowed to crawl/fly up the vial for 10 s. The number of adult flies crossing the 8 cm distance in 10 s was counted. The assay was repeated three times with a 1-min rest between readings. Flies were assayed again at 14-days and 21-days. Each genotype was assayed in triplicate. Statistical significance was calculated with Microsoft Excel using a two-tailed t-test comparing *Mef2* > *mCherry* control RNAi to each experimental RNAi to calculate *p* values. The average, standard deviation calculations, and graphs were conducted using Graph Pad Prism.

### Micro Computed Tomography

Pupae or one-week-old aged-matched adults were stained and imaged as previously described (Schoborg et al., 2019; Schoborg, 2020). In short, adults were washed in 1x phosphate-buffered saline +0.5% Triton-X 100 (0.5% PBST) for 5 min. Flies were then fixed in 1 ml Bouin’s solution (5% acetic acid, 9% formaldehyde, 0.9% picric acid) for 24 h and washed 3X in 1 ml wash buffer (0.1 M Na_2_HPO_4_, 1.8% sucrose) and stained with 1 ml of 0.1 N solution of Lugol’s solution (100 mg/ml potassium iodide, 50 mg/ml iodine) for 2–3 days. Post-staining, the flies were washed and stored in water at room temperature until scanning. For pupal imaging, the samples were heated to 95°C in 1 ml of 0.5% PBST for 30 s in a heat block. The pupae were then fixed in Bouin’s solution for 2–3 days. After washing 3X in wash buffer, holes were poked at the anterior and posterior end of the pupal case using a microdissection needle cautiously as to not harm the pupal tissue. Fixed pupae were stained with Lugol’s solution similar to adults. Samples were mounted in a pipette tip filled with water and attached to the μCT sample holder using silicone and parafilm. A single fly was inserted headfirst into the pipette tip and pushed gently until secure within the pipette tip. Silicone wax was used to seal the opening of the pipette tip to avoid evaporation. Samples were scanned using a Bruker SKYSCAN 1172 desktop scanner (Bruker Corp, Billerica, MA, United States) equipped with a Hamamatsu 10 megapixel x-ray camera (detector) with an 11.54 μm pixel size (Hamamatsu Corp, Bridgewater, NJ, United States). Scans were performed at 40 kilovolts (kV), 110 microamps (μA) and 4 watts (W) of power. Small camera settings at a resolution of 1.15 μm with 360° of sample rotation and 3,000 ms exposure time were used for acquisition. Acquisition for a single fly took overnight for this resolution. The random movement was set to 10 and frame averaging ranged from 5–8. NRecon software (Bruker MicroCT, v1.6.10.1) was used for reconstructing the tomographs. The reconstruction process was optimized for misalignment compensation and ring artifact reduction settings, separately for each data set. Reference scans were used to account for sample movement during scanning. Beam hardening was set to 0%.

### Adult and Pupal Thoracic Muscle Measurements

For μCT analysis, for each genotype, a single representative cross-section from three animals was analyzed using muscles and gut approximating to the same size. The area of eight dorsal median indirect flight muscles (four on each side) (adults) or twelve dorsal median indirect flight muscles (six on each side) (pupae), ventral to dorsal, was calculated by outlining each muscle using FIJI and calculating the area in arbitrary units. For each violin plot, the area of each region of interest (individual muscles) is represented by a single dot and the median and quartiles are represented by the horizontal solid lines. The graph was generated using GraphPad Prism. The significance was determined using GraphPad Prism using an unpaired two-tailed t-test with Welch’s correction comparing control (*Mef2* GAL4 > *mCherry* RNAi) to each experiment. For sarcomere size analysis, 20 sarcomeres per muscle were randomly selected through multiple Z sections. The length and width were measured using FIJI (Schindelin et al., 2012). Care was taken to ensure the maximum width was measured for genotypes with disorganized myofibrils.

### Immunofluorescence

For skeletal muscle, the thoraces of week-old adults or pupae removed from their pupal case were dissected in 1x phosphate buffered solution (PBS). After the head and abdomen were removed, the intact thoraces were fixed in 4% Paraformaldehyde (PFA) in Grace’s media for 1 h then rinsed in antibody (Ab) wash (1x PBS, 0.1% Triton X-100x, 0.1% bovine albumin). The thoraces were rinsed 1x in 1x PBS. Using small surgical scissors, each thorax was bisected in 1x PBS, then permeabilized in Ab wash +0.5% Triton-X 100 for 5 min. After 3x 10 min washes in Ab wash, the bisected thoraces were incubated in primary antibody at 4 °C overnight. After 3x 10 min washes, secondary antibody was added for several hours at room temperature in Ab wash, goat antimouse IgG2b Alexa 488 was added for several hours at room temperature. Samples were washed again with Ab wash 3x 10 min, then mounted in Vectashield (Vector Laboratories Cat# H-1000, RRID:AB_2336789). For fly hearts, females of the appropriate genotypes were fattened on wet yeast paste. After 1 week, the ovaries and gut were removed in Artificial *Drosophila* Hemolymph [ADH; 108 mM NaCl, 5 mM KCl, 2 mM CaCl_2_, 8 mM MgCl_2_, 1 mM NaH_2_PO_4_, 4 mM NaHCO_3_, 10 mM sucrose, 5 mM trehalose, and 5 mM HEPES (pH 7.1)]. Using small surgical scissors, two incisions were made along the lateral sides of the abdomen and the interior of the abdomen was further cleaned of debris cautiously as to not disturb the dorsal tube. The tissues were fixed for 20 min in 4% PFA in Grace’s media. Primary and secondary antibody was added as for thoraces. For actin, Rhodamine conjugated Phalloidin or Alexa Fluor 488 Phalloidin (1:200, Thermo Fisher Scientific, Waltham, MA, United States) was used. For mitochondrial labeling, anti-ATP synthase (Abcam, Cat # ab14748, RRID:AB_301447) was used 1:500. Secondary antibody was anti-mouse IgG2b Alexa 488 (1:500, Jackson ImmunoResearch Laboratories, Inc., West Grove, PA). For the main figures, Z-stacks were taken using a Zeiss 700 confocal and 63X 1.4 NA objective with minimal optimal sectioning depth. For Supplementary Figure S10, images were taken using a Nikon A1R confocal microscope using 10X, 0.45 NA. and 40X, 1.3 NA objectives. Z-stacks at 1 µm intervals were collected and images are presented as maximum intensity projections encompassing the dorsal half of the heart.

### Intravital Fluorescence Microscopy

Intravital fluorescence imaging of adult hearts was carried out as previously described (Petersen et al., 2020). To image the heart, control or experimental RNAi was expressed using 4xHand GAL4 in a background of tdTomato expression under control of the cardiomyocyte-specific R94C02 enhancer (CM-tdTomato) (Klassen et al., 2017). Adult flies were briefly anesthetized with CO_2_ and adhered dorsal side down to a glass coverslip using Norland Optical Adhesive (Norland Products, Cranberry, NJ, United States) that was then cured with a 48-watt UV LED light source (LKE) for 60 s. Animals were allowed to recover for 10 min prior to imaging. The adult hearts were imaged through the dorsal cuticle at a rate of 200 frames per second (fps) for 10 s using an ORCA-Flash 4.0 V3 sCMOS camera (Hamamatsu Corp., Bridgewater, NJ, United States) on a Nikon Ti2 inverted microscope controlled with Nikon Elements software (Nikon Inc., Mellville, NY, United States). Excitation light at 550 nm was provided by a Spectra-X Light Engine illuminator (Lumencor, Inc., Beaverton, OR, United States) and emission was collected through a 555–635 band-pass filter. To generate M-mode kymographs, a 1-pixel wide line was drawn through the heart chamber in the A2 segment, and the fluorescence intensity along this line for the full-time course was plotted using ImageJ. End-diastolic dimensions (EDD) and end-systolic dimensions (ESD) were calculated from the processed M-mode traces by manually measuring the distance between the heart walls at full relaxation and full constriction, respectively. An average of five measurements of EDD and ESD were obtained from each trace. Heart rate was calculated by manually counting the number of systoles over 10 s. Fractional shortening (FS) was calculated as [(EDD-ESD)/EDD] × 100. Heart periods were calculated from an average of five measurements made on processed M-mode traces calculated by manually measuring the time between systoles. Calculation of heart period (HP) median and standard deviation (SD) were determined using GraphPad Prism software. Arrhythmicity index was calculated as HP SD/HP median. All statistical analysis was performed using GraphPad Prism software and analyzed by One-way ANOVA followed by Tukey’s multiple comparisons test. Differences were considered statistically significant at *p* < 0.05.

### ATP

ATP assays were performed as previously described (Sen et al., 2016). In short, about 20 thoraces from pupae or adult flies were homogenized in 75 µl of ATP extraction buffer (100 mM Tris-Cl, pH 8.0, 4 mM EDTA, pH 8.0, 6 M guanidine hydrochloride) and used for both ATP and bicinchoninic acid (BCA) assay. ATP concentrations were determined using ATP determination Kit (Invitrogen, Thermo Fischer Scientific, Waltham, MA, United States) as per the manufacturer’s protocol. The luciferase activity was measured in 96-well format using a CLARIOstar plate reader (BMG Labtech Inc, Cary, NC, United States). Protein concentrations were determined using a BCA protein assay kit. 5 µl of diluted samples (1:25) were mixed with 100 µl of BCA reagent (Pierce BCA protein assay Kit, Thermo Fischer Scientific, Waltham, MA) and protein concentrations were measured on a CLARIOstar plate reader (BMG Labtech Inc, Cary, NC, United States). Results were averaged over three technical replicates from each samples and represented as percent ATP concentrations normalized to the protein concentrations.

## Results

### Reduced *Rswl*, *Scu* and *Mldr* in Muscle Affects Eclosion

We previously showed that loss of *rswl*, *scu* and *mldr* is lethal which makes it difficult to study adult tissue-specific phenotypes that arise from loss of mtRNase P (Sen et al., 2016; Saoji et al., 2021). Using the *Drosophila* UAS/GAL4 system we showed ubiquitous expression of *mldr* ^*KK*^, *rswl* ^*GD*^ and *scu* ^*GL*^ RNAi phenocopies our protein null mutants with the resulting larvae pupating but with no or significantly reduced adult emergence from the pupal case, which is called eclosion (Sen et al., 2016). Using qPCR, we also showed *Actin* GAL4 > *mldr* ^*KK*^, *rswl* ^*GD*^ and *scu* ^*GL*^ substantially reduced mRNA levels (Saoji et al., 2021).

Here we model myopathies associated with mitochondrial disease by conditionally expressing RNAi in skeletal muscle. *Myocyte Enhancing Factor* (*Mef*) *2* GAL4 is expressed during embryogenesis and in the muscle precursor cells that will form the adult muscles (Baker et al., 2005; Ranganayakulu et al., 1995). We examined *mtRNase P* RNAi knockdown at two temperatures [room temperature (RT) and 29°C]. Higher temperatures increase RNAi effectiveness and thus supply us with graded phenotypes (Kaya-Çopur and Schnorrer, 2019). To confirm that these RNAi strains also result in lower protein levels, we used western blotting to compare adult heads (no GAL4 expression) vs. thorax (*Mef2* GAL4 expression) for each protein. *Mef2* GAL4 reduced the level of Rswl, Scu and Mldr in the muscle-rich thorax for all the RNAi lines examined (Supplementary Figure S1). This reduction was greater in males, which was not unexpected as RNAi is known to function more efficiently in males (Dietzl et al., 2007). In addition, we performed qPCR on female thoraces (Supplementary Figure S2). mRNA levels are reduced in *Mef2* > *rswl* ^*GD*^, *scu* ^*HMS*^_*,*_ and *mldr* ^*KK*^. *Mef2* > *scu* ^*GL*^ did not show a reduction in mRNA despite having reduced protein levels.

Eclosion of adult flies is an energy-intensive event (Merkey et al., 2011). *Mef2* GAL4 knockdown of *rswl*, *scu,* and *mldr* at RT and 29°C resulted in normal pupation rates compared to *GAL4* and *mCherry* controls (Figures 1B,C,E,F). However, eclosion rates for all three were reduced with many exhibiting pharate lethality where the animals die either in the pupal cases that are fully developed, or partially emerged from their pupal cases (Figures 1B,C,E,F). Thus, it appears pupation happened at a normal rate and many aspects of pupal development occur, but eclosion failed late in pupal development. At RT, no *Mef2* > *rswl* ^*GD*^ pupae eclosed and 79% were pharate lethal. Although a small percentage (8.7%) of *Mef2* > *scu* ^*HMS*^ pupae eclosed, the rest were pharate lethal. At 29°C, *Mef2* > *rswl* ^*GD*^ pupae failed to develop and no *Mef2* > *scu* ^*HMS*^ eclosed, with all the resulting pupae pharate lethal. In addition, at 29°C, 66% of *Mef2* > *mldr* ^*KK*^ pupae failed to eclose; 34% did not develop past pharate lethality. Even though the majority ecloses, most of the adults died 5 days after eclosion (Figure 1D). These results show that knocking down all *Drosophila* mtRNase P subunits in muscle causes severe problems with eclosion. We also monitored survival for 30 days (Supplementary Figure S3). At RT, there was no difference in fly survival. At 29°C, *Mef2* > *scu* ^*GL*^ females and males died faster, as did *Mef2* > *mldr* ^*KK*^ females.

### Reduction of mtRNase P Subunits in Muscle Reduces Muscle Size, Integrity, and Function

**FIGURE 2:**
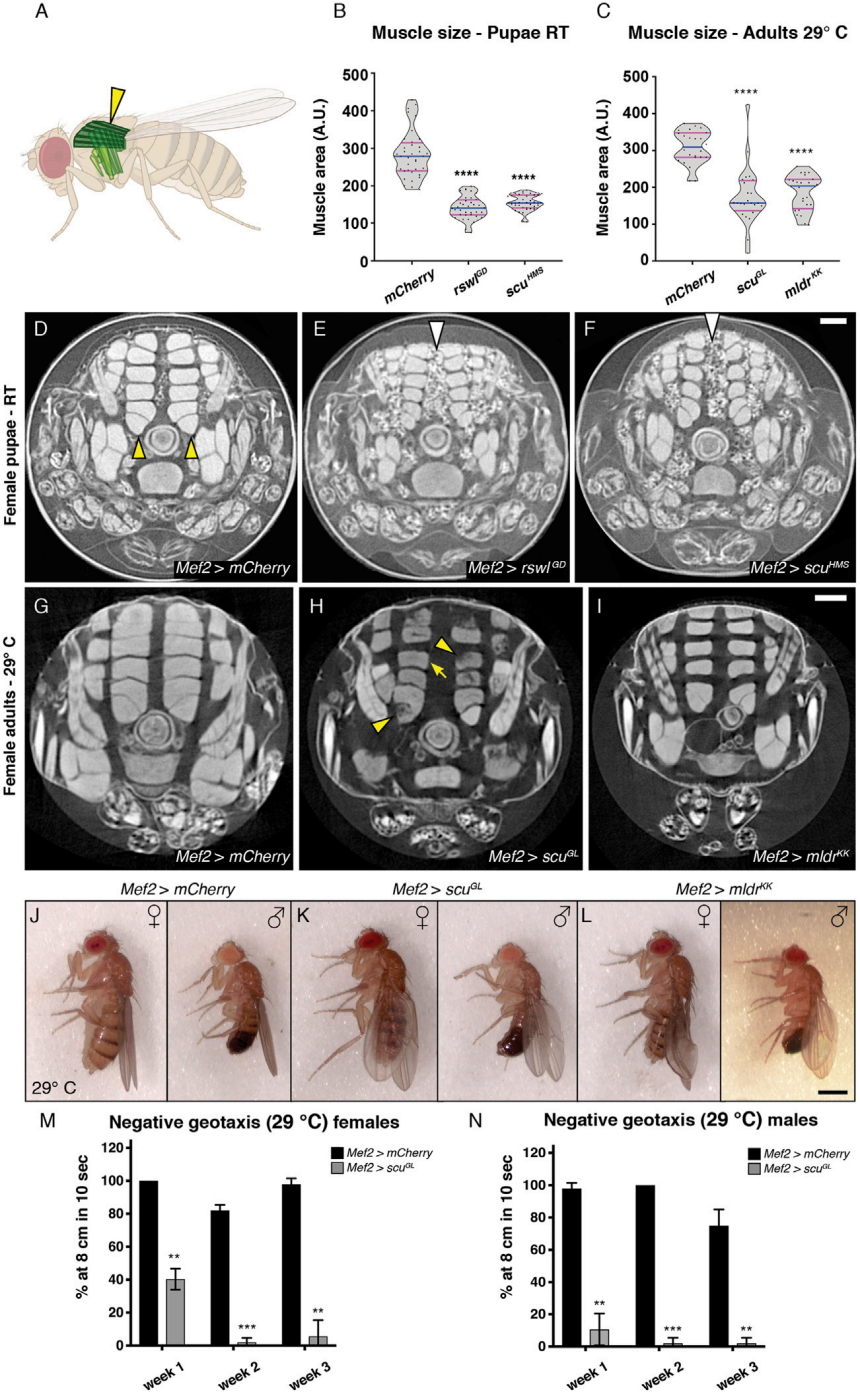
Reduction of *rswl*, *scu,* and *mldr* in skeletal muscle decreases muscle size and function. **(A)** Fly schematic of the dorsal longitudinal muscles analyzed (dark green and yellow arrowhead). **(B,C)** Quantification of muscle size in female pupae at RT **(B)** and adult females at 29°C **(C)**. Each point on the violin plot represents a single dorsal longitudinal muscle measured from a cross-section of three different animals per genotype. The median and quartiles are represented by blue and magenta horizontal solid lines, respectively. Further description can be found in the materials and methods. **(D–F)** μCT analysis of female pupae grown at room temperature (RT) expressing *rswl* ^*GD*^ RNAi **(E)** and *scu* ^*HMS*^ RNAi **(F)** using *Mef2* GAL4. The dorsal longitudinal muscles are smaller **(E,F)** compared to *mCherry* control [**(D)**, yellow arrowheads]. There is also more white globular material between the muscles with RNAi [**(E,F)**, white arrows] compared to control **(D,G–I)** μCT analysis of indirect flight muscle size in adult females grown at 29 °C. **(G)** *Mef2* > *mCherry* control flies have intact dorsal longitudinal muscles. **(H, I)** *Mef2* > *scu* ^*GL*^ and *Mef2* > *mldr* ^*KK*^ expressing adults have smaller dorsal longitudinal muscles [arrow, **(H)**]. *Mef2* > *scu* ^*GL*^ adults also exhibit apparent muscle tears and gaps [**(H)**, arrowheads] **(J–L)** Adults expressing *scu* ^*GL*^ **(K)** and *mldr* ^*KK*^ **(L)** RNAi using *Mef2* GAL4 display droopy and crumpled wings compared to control *mCherry* RNAi at 29°C **(J)**. Fraction with phenotype: **(J)** *n* = 0/112 females, 1/76 males, **(K)** *n* = 54/54 females, 23/26 males, **(L)** *n* = 118/118 females, 19/19 males. **(M,N)** Negative geotaxis ability decreases with age in *Mef2* > *scu* ^*GL*^ adult females **(M)** and males **(N)** at 29°C. **(M,N)** Error bars = standard deviation. Statistical significance was calculated with Microsoft Excel using a two-tailed t-test comparing *Mef2* > *mCherry* control RNAi to each experimental RNAi to calculate *p* values. ** = *p* < 0.01, *** = *p* < 0.001, **** = *p* < 0.0001. Graphs were plotted using GraphPad Prism. Scale bar = 100 μm in F for **(D–F)** and I for **(G–I)**, 1.25 mm in L for **(J–L)**.

Since *mtRNase P* RNAi knockdown is muscle specific using *Mef2* GAL4, and since reduction of each subunit led to a substantial amount of pharate lethality, we examined muscle integrity and size using micro computed tomography (μCT). None of the *Mef2* > *rswl* ^*GD*^ animals eclosed at room temperature (Figures 1B,E). Using *Mef2* > *mCherry* as a control, we analyzed pupae that were grown at RT for muscle defects. *Mef2* > *mCherry* pupae had the expected array of dorsal longitudinal muscles (DLM) (Figures 2A,D, yellow arrowheads). Both *Mef2* > *rswl* ^*GD*^ and *Mef2* > *scu* ^*HMS*^ pupae also had the normal array of DLMs with no apparent tears or gaps indicating general muscle development proceeded normally (Figures 2E,F). However, these muscles were significantly smaller compared to control (Figure 2B). Driving *rswl* ^*GD*^ RNAi expression more strongly and ubiquitously using *Tubulin* GAL4 led to disorganized muscles (Supplementary Figure S5). In addition, *rswl* ^*GD*^ and *scu* ^*HMS*^ RNAi pupae had excess white globular material between the muscles which was also apparent while using confocal microscopy (Figures 2E, F, arrowheads, Supplementary Figure S4).

We also examined adult flies for muscle defects. Using μCT analysis, we examined adult flies reared at 29°C and found *Mef2* > *scu* ^*GL*^ and *Mef2* > *mldr* ^*KK*^ flies had smaller DLMs compared to control, similar to the pupae (Figures 2C,G–I). While *Mef2* > *mldr* ^*KK*^ adults had intact muscles, *Mef2* > *scu* ^*GL*^ adults exhibited gaps or tears in their muscles (Figure 2H, arrowheads). Many mitochondrial myopathies present with muscle weakness and ataxia (Pfeffer and Chinnery, 2013). We observed that *Mef2* > *scu* ^*GL*^ and *mldr* ^*KK*^ adult flies had defective wings after eclosion (Figures 2J–L). Compared to *mCherry* control flies (Figure 2J), *Mef2* > *scu* ^*GL*^ females and males displayed drooped wings (Figure 2K, Supplementary Figure S5) whereas *mldr* ^*KK*^ displayed crumpled or drooped wings at 29°C, but normal wing posture at RT (Figure 2L, Supplementary Figure S6). This abnormal wing posture is often indicative of indirect flight muscle defects; however, the crumpled wings could be due to the role of Mef2-responsive cells in wing-heart formation (Nongthomba and Ramachandra, 1999; Tögel et al., 2008). In addition to decreased muscle size and defective wing posture, muscle-specific *mtRNase P* knockdowns also translated into locomotive abnormalities. Both *Mef2* > *scu* ^*GL*^ and *Mef2* > *mldr* ^*KK*^ exhibited an age-progressive decrease in climbing ability (Figures 2M,N, Supplementary Figure S6). These muscle phenotypes demonstrate that mtRNase P is critical for muscle growth during pupal development and function during adulthood and that muscle function continues to decline with age.

### Reduction of mtRNase P Subunits in Muscle Disrupts Myofibril Organization and Mitochondrial Morphology

**FIGURE 3:**
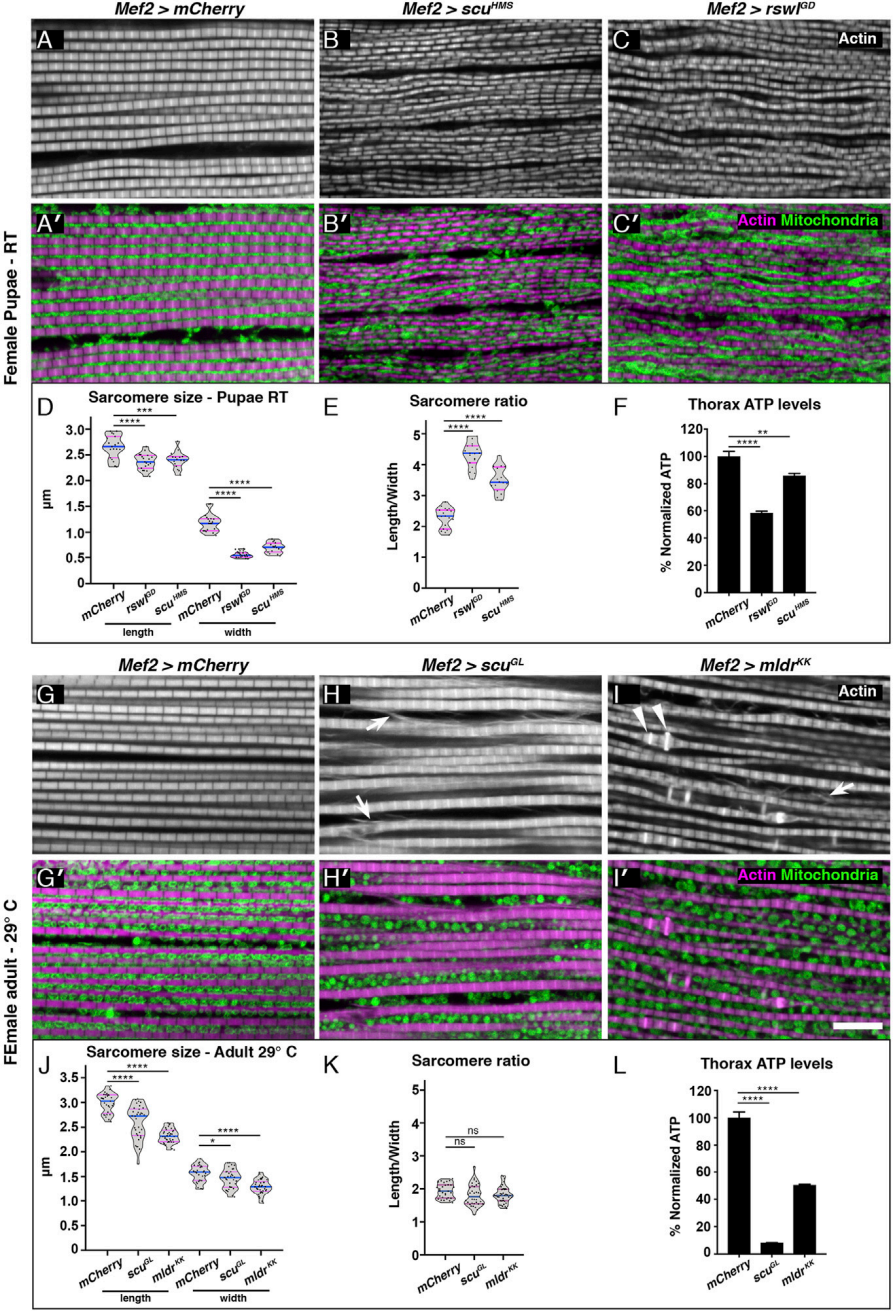
Reduction of mtRNase P subunits in skeletal muscle causes smaller sarcomeres, and myofibrillar disorganization and disrupts mitochondrial morphology. **(A–C′)** Confocal microscopy micrographs of thoracic skeletal muscle isolated from female pupae grown at room temperature (RT). *Mef2* > *mCherry* control muscle exhibits a regular sarcomere pattern [**(A)**, white, **(A′)**, magenta] and mitochondria are tightly packed and regularly spaced between the myofibrils [**(A′)**, green]. *Mef2* > *scu* ^*HMS*^ and Mef2> *rswl* ^*GD*^ pupae have disorganized myofibrils [**(B,C)**, white] and mitochondria are not evenly distributed between the myofibrils nor regularly shaped compared to control [**(B′,C′)**, green vs. **(A,A′)**]. **(D)** *Mef2* > *scu* ^*HMS*^ and *rswl* ^*GD*^ pupae have shorter, thinner sarcomeres compared to *mCherry* control resulting in a larger length/width ratio **(E, F)** ATP levels in isolated thoraces from female pupae grown at RT. **(G–I′)** Skeletal muscle isolated from adult females grown at 29°C. **(G)** *Mef2* > *mCherry* control muscle exhibits a regular sarcomere pattern [**(G)**, white, **(G′)**, magenta] and mitochondria are evenly distributed between the myofibrils and are uniform in size and shape [**(G′)**, green]. *Mef2* > *scu* ^*GL*^ and *Mef2*> *mldr* ^*KK*^ adults have disorganized myofibrils [**(H,I)**, white] that exhibit actin abnormalities (arrows, arrowheads). In addition, mitochondria are less tightly packed **(H′,I′)** and not evenly distributed between the myofibrils **(H′,I′)** compared to control **(A,A′)**. **(J)** *Mef2* > *scu* ^*GL*^ and *Mef2*> *mldr* ^*KK*^ adults have shorter, thinner sarcomeres compared to *mCherry* control but retain the same length/width ratio as control **(K)**. **(L)** ATP levels in isolated thoraces from adults grown at 29°C. Actin = white **(A–C,G–I)**, magenta **(A′-C′,G′-I′)**. anti-ATP synthase = green **(A′-C′,G′-I′)**. **(D,E,J,K)** Each point on the violin plot represents an average of 20 measurements of one muscle from eight to ten different animals per genotype. The median and quartiles are represented by blue and magenta horizontal solid lines, respectively. Further description can be found in the materials and methods. Error bars = standard deviation. Statistical significance was calculated with GraphPad Prism using a two-tailed t-test comparing *Mef2* > *mCherry* control RNAi to each experimental RNAi to calculate *p* values. * = *p* < 0.05, ** = *p* < 0.01, *** = *p* < 0.001, **** = *p* < 0.0001. Graphs were plotted using GraphPad Prism. Scale bar = 10 μm in **(I′)** for **(A–C′)** and **(G–I′)**.

Since mtRNase P RNAi knockdown with *Mef2* GAL4 caused smaller muscles and decreased locomotion, we examined sarcomere size and mitochondrial morphology (Figure 3). *Mef2* > *mCherry* control pupae had a regular array of myofibrils with evenly spaced mitochondria (Figures 3A, A′). In contrast, *Mef2* > *rswl* ^*GD*^ and *scu* ^*HMS*^ DLMs exhibited disorganized, wavy myofibrils (Figures 3B–C′). We measured the sarcomere length and width and determined that both genotypes had statistically shorter and narrower sarcomeres which caused a higher length/width ratio compared to *mCherry* control (Figures 3D, E). In addition, the mitochondria were not evenly spaced between the myofibrils and had abnormal morphology compared to control (Figures 3B′, C′). *Mef2* > *rswl* ^*GD*^ and *scu* ^*HMS*^ thoraces also had reduced ATP levels (Figure 3F). These phenotypes support that reduced mtRNase P function during muscle development causes myofibril and mitochondrial defects.

We also examined myofibril organization and mitochondrial morphology in adults. *Mef2* > *mCherry* control animals had regularly spaced myofibrils and evenly distributed mitochondria (Figures 3G,G′). In contrast, *Mef2* > *scu* ^*GL*^_*,*_ and *mldr* ^*KK*^ DLMs had disorganized and wavy myofibrils similar to what we observed in pupae. In addition, the actin labeling indicated the myofibrils were shearing or tearing (Figures 3H, I, arrows). Myofibrils in *Mef2* > *mldr* ^*KK*^ animals also had abnormal actin accumulation in a subset of the sarcomeres (Figure 3I, arrowheads). As with pupae, the sarcomeres were statistically shorter and narrower compared to control, but not to the same degree (Figure 3J). Furthermore, the sarcomeres were smaller proportionally, in contrast to pupae (Figure 3K). Mitochondrial morphology was also altered in *Mef2* > *scu* ^*GL*^ and *mldr* ^*KK*^ compared to control (Figures 3H′,I′, Supplementary Figure S7). For *Mef2* > *scu* ^*GL*^, mitochondria were rounder and not evenly spaced (Figure 3H′). Furthermore, we noticed *Mef2* > *scu* ^*GL*^ had three different phenotypes (Supplementary Figure S7). One phenotype showed highly disrupted and torn myofibrils (Supplementary Figure S7B) which could correspond to the muscle gaps indicated with μCT analysis (Figure 2H). These muscles had highly disrupted mitochondrial morphology (Supplementary Figure S7B’). In addition, some muscles had very sparse mitochondria (Supplementary Figure S7C’). *Mef2* > *mldr* ^*KK*^ animals did not have tightly packed, regularly distributed mitochondria (Figure 3I′). As with pupae, ATP levels were reduced in both genotypes (Figure 3L). These data together support that reduced mtRNase P function in muscle causes myofibrillar disorganization and altered mitochondrial morphology and function.

### Heart-specific Reduction of mtRNase P Disrupts Heart Myofibril Organization and Mitochondrial Morphology

**FIGURE 4:**
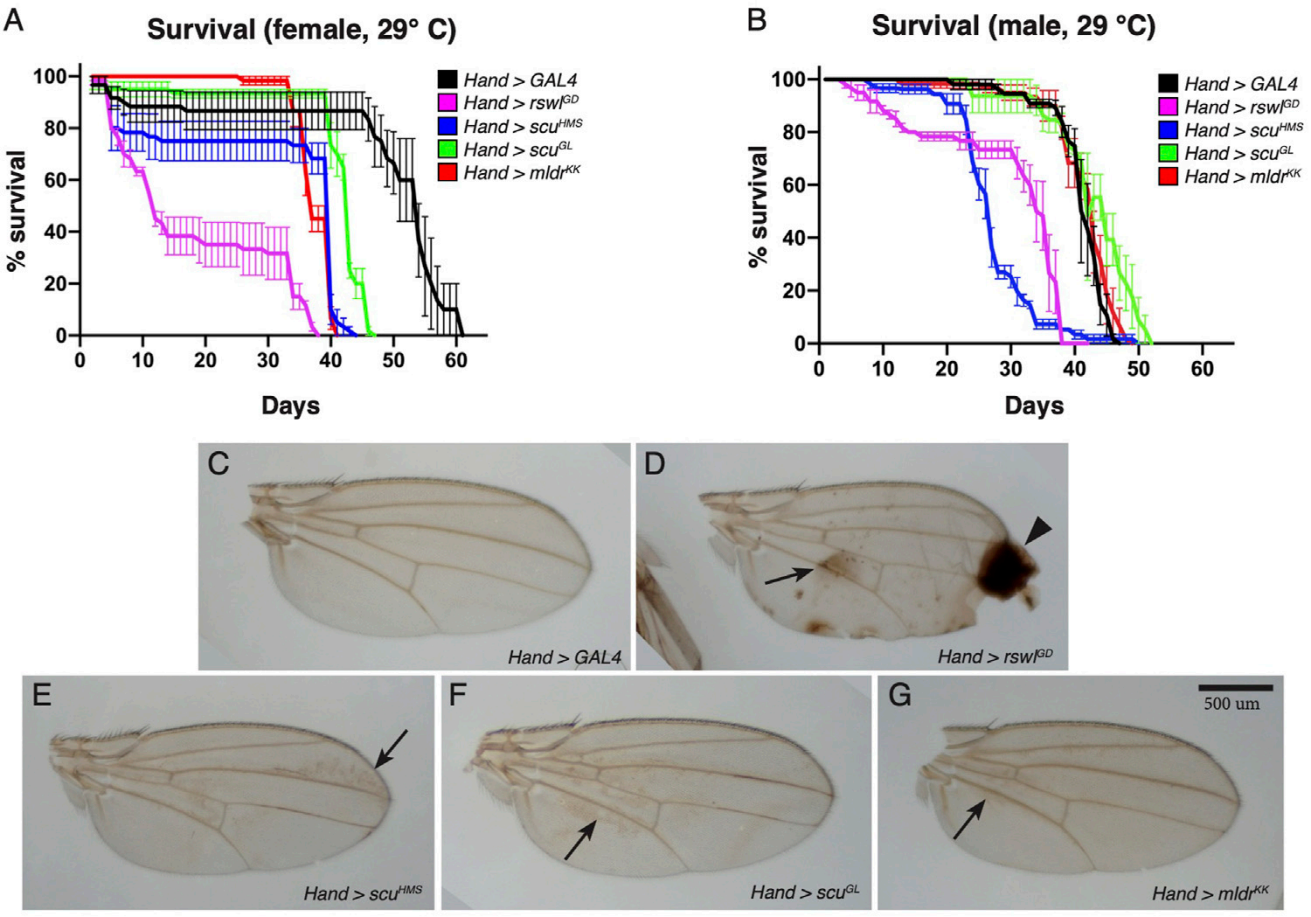
Reduction of mtRNase P subunits in the heart reduces lifespan and causes wing defects. **(A,B)** Lifespan measurements for adults grown at 29°C expressing *mtRNase P* RNAi using *Hand* GAL4. For females **(A)**, all RNAi genotypes have statistically shortened lifespans compared to *GAL4* RNAi control (Bonferroni *p* value = 0). For males **(B)**, *rswl* ^*GD*^ RNAi (Bonferroni *p* value = 0) and *scu* ^*HMS*^ RNAi (Bonferroni *p* = 0) have statistically shortened lifespans compared to *GAL4* RNAi control. **(C–G)** Representative wings from *Hand* > *GAL4*, *rswl* ^*GD*^, *scu* ^*HMS*^, *scu* ^*GL*^_*,*_ and *mldr* ^*KK*^ female flies grown at 29 °C. *mtRNase P* RNAi wings have brown discoloration [**(D–G)**, arrows] compared to *GAL4* RNAi control **(C)**. *Hand* > *rswl* ^*GD*^ RNAi wings also have blistering and scarring [**(D)**, arrowhead]. **(A,B)** Error bars = s.e.m. The graphs were plotted using GraphPad Prism. The significance between the survival distributions was done using the Log-Rank test between the combined triplicates using OASIS 2. More details can be found in the materials and methods. Scale bar = 500 μm in G for **(C–G)**.

Cardiomyopathy is a common symptom of mitochondrial disease (El-Hattab and Scaglia, 2016). To determine how mtRNase P reduction affects heart function in *Drosophila*, we expressed *rswl*, *scu*, and *mldr* RNAi under control of heart specific *4xHand* (*Hand*) GAL4 (Zhu et al., 2017). Unlike the *Mef2* GAL4 results, none of the *Hand* > RNAi experimental genotypes caused a delay or decrease in pupation or eclosion rates (Supplementary Figure S8). While pupation and eclosion were normal, we found there was a difference in lifespan with *mtRNase P* RNAi (Figures 4A, B, Supplementary Figure S8). *Drosophila* contain unique structures called wing hearts that express *Hand* and originate from cardiac mesoderm in the embryo (Tögel et al., 2008). Wing hearts are responsible for ensuring normal hemolymph flow through the narrow wings and disruption to wing hearts causes wing defects (Tögel et al., 2008, 2013). In addition to reduced lifespan with *mtRNase P* knockdowns, we also noticed wing defects (Figures 4C–G, Supplementary Figure S9). Normal wings should be clear as we saw with control *Hand* > *GAL4* RNAi flies (Figure 4A). In contrast, *Hand* > *rswl* ^*GD*^, *scu* ^*HMS*^, *scu* ^*GL*^ and *mldr* ^*KK*^ exhibited brown areas on the wings (Figures 4D–G, arrows). *Hand* > *rswl* ^*GD*^ had the most severe defects, with the wings often blistered (Supplementary Figure S9). These data support that mtRNase P knockdown with *Hand* GAL4 reduces lifespan and affects cardiac-derived wing heart function.

**FIGURE 5:**
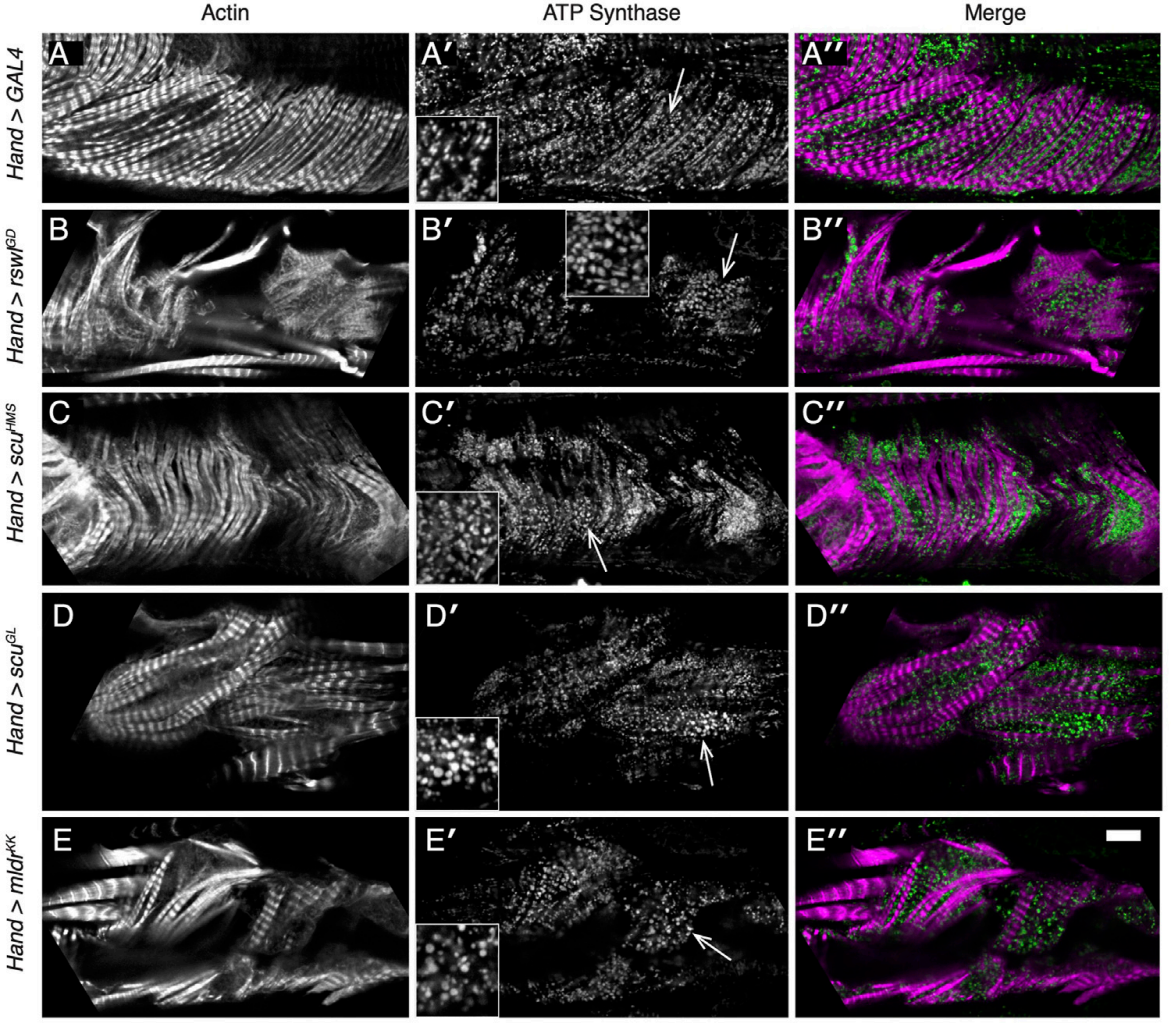
Reduction of mtRNAse P subunits in the adult heart disrupts myofibril organization and mitochondrial morphology. **(A–E′′)** Confocal microscopy micrographs of hearts from adult female flies grown at 29°C. **(A–A′′)** Mitochondria [**(A′)**, green] in control *Hand* > *GAL4* RNAi hearts form a lattice between the regularly spaced myofibrils [**(A′)**, magenta]. **(B-E′′)** Labeled hearts from flies expressing *mtRNase P* RNAi using *Hand* GAL4 indicates the myofibril pattern is disrupted [**(B–E)**, white, **(B′′–E′′)**, magenta]. Mitochondrial morphology is also different compared to control with many mitochondria larger than control [**(B′–E′)**, arrows vs. **(A′)**]. The area of the inset is indicated by the arrow. Actin = white, **(A–E)**, magenta **(A′′–E′′)**. anti-ATP synthase antibody = white **(A′–E′)**, magenta **(A′′–E′′)**. Scale bar = 10 μm in E″ for **(A–E′′)** and 5 μm for insets.

We also examined heart structure and heart mitochondria using immunofluorescence (Figure 5, Supplementary Figure S10). Adult flies with *mtRNase P* RNAi had disorganized myofibrillar structure compared to *GAL4* RNAi control (Figures 5B–E vs. Figure 5A, Supplementary Figure S10). Mitochondrial morphology in control *Hand* > *GAL4* animals indicated the organelles were mostly evenly dispersed between the myofibrils (Figures 5A′,A"). *Hand* > *rswl* ^*GD*^, *scu* ^*HMS*^, *scu* ^*GL*^, and *mldr* ^*KK*^ adults had very disorganized myofibrils, thus, mitochondria could not be evenly dispersed. In addition, mitochondria appear larger compared to *GAL4* control (B′-E′, arrows).

### Reduction of *Rswl* and *Mldr* in the Heart Impairs Heart Contractility

**FIGURE 6:**
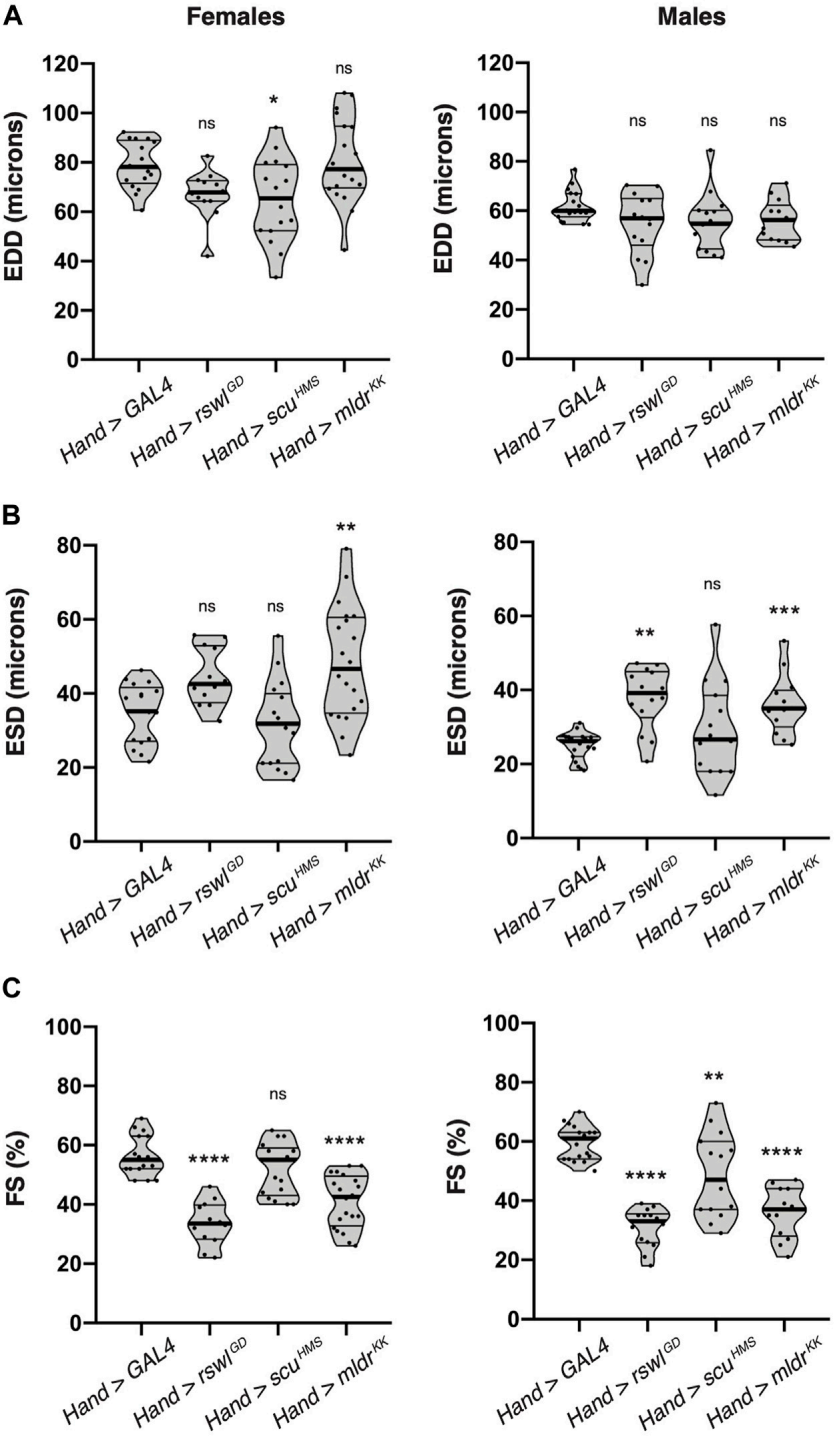
Reduction of mtRNAse P subunits in the adult heart significantly reduces heart contractility. Intravital imaging kymographic heart measurements for **(A)** end-diastolic dimension (EDD), **(B)** end-systolic dimension (ESD), and **(C)** percent fractional shortening (FS) from two-day-old female and male adult hearts expressing *GAL4* (control), *rswl* ^*GD*^, *scu* ^*HMS*^, and *mldr* ^*KK*^ RNAi using *Hand* GAL4 at 29°C. The points on each violin plot represent an average of five measurements from one fly and the median and quartiles are represented by the horizontal solid lines. Statistical significance was calculated with GraphPad Prism using Ordinary one-way ANOVA and Tukey’s multiple comparison test. *p* values compare to control (*GAL4*) to each experimental RNAi. * = *p* < 0.05, ** = *p* < 0.01, *** = *p* < 0.001, **** = *p* < 0.0001. ns = not significant. The graphs were plotted using GraphPad Prism.

Because heart-specific reduction of mtRNase P caused a decrease in lifespan, wing defects, as well as defects in a myofibrillar organization and mitochondrial morphology, we examined heart function using intravital fluorescence microscopy. The *Drosophila* heart is a relatively simple dorsal tube running the length of the animal (Supplementary Figure S11). Even though insects have an open circulatory system, the *Drosophila* dorsal tube shares many characteristics with hearts in higher organisms and has been used to model heart disease (Taghli-Lamallem et al., 2016). To analyze heart contractility, we used kymographic data to measure the end-diastolic dimensions (EDD) and end-systolic dimensions (ESD) of two-day-old males and females expressing *rswl*, *scu,* and *mldr* RNAi under the control of *Hand* GAL4 at 29°C (Figure 6, Supplementary Figure S11). We examined 2-day-old flies to observe the first heart deficiencies after eclosion. This also enabled us to decrease fly to fly variation in the data and to avoid measuring heart function in very sick flies. Fractional shortening (FS) is defined as the ratio of the difference between EDD and ESD over EDD measurements (Supplementary Figure S11). Decreases in fractional shortening are indicative of weak contractility (Wolf, 2012; Mathew et al., 2017). The EDD for *mtRNase P* RNAi was significantly reduced in *Hand* > *scu* ^*HMS*^ females (Figure 6A). For ESD, by contrast, *Hand* > *scu* ^*HMS*^ had no statistically different effect. Males, however, exhibited increased ESD when *rswl* and *mldr* were reduced (Figure 6B). This increase in ESD indicates a reduction in the displacement of the heart walls with each contraction, consistent with weak contractility. Indeed, fractional shortening was greatly diminished in both males and females with loss of *rswl* and *mldr*, with males sensitive to reduction of *scu* as well (Figure 6C). Thus, reduction of *rswl* had the greatest effect in general, consistent with *Hand* > *rswl* ^*GD*^ significantly decreasing lifespan. *Hand* > *scu* ^*HMS*^ had a significantly shorter lifespan but not as great an effect on fractional shortening, however, these flies did not start dying as quickly as *rswl* ^*GD*^ (Figures 4E, F). Surprisingly, although *Hand* > *mldr* ^*KK*^ also had significantly decreased fractional shortening and disrupted myofibril structure, these flies had normal lifespans (Figures 4E, F). These data suggest that cardiac defects are observed soon after eclosion and that reduction of *rswl* and *mldr* has the greatest effect on heart contractility.

### Reduction of *Rswl* and *Mldr* in the Heart Greatly Increases Heart Arrhythmia

**FIGURE 7:**
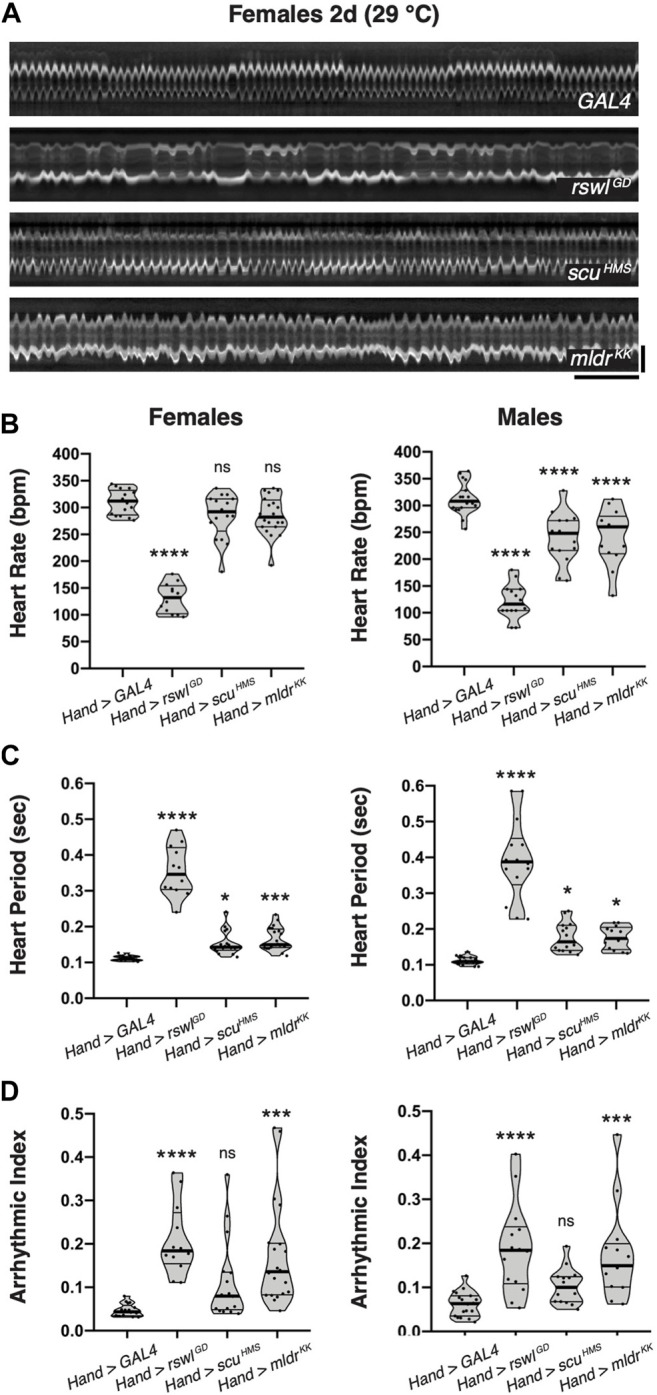
Reduction of *rswl* and *mldr* in the adult heart greatly increases heart arrhythmicity. **(A)** Representative kymographs from 2-day-old females expressing *GAL4*, *rswl* ^*GD*^, *scu* ^*HMS*^_*,*_ or *mldr* ^*KK*^ RNAi in the heart using *Hand* GAL4 at 29°C. Kymographic analysis of heart rate (BPM = beats per minute) **(B)**, heart period **(C)**, and arrhythmicity index **(D)** for females and males at 29°C. The points on each violin plot represent measurements from one fly and the median and quartiles are represented by the horizontal solid lines. Statistical significance was calculated with GraphPad Prism using Ordinary one-way ANOVA and Tukey’s multiple comparison test. *p* values compare to control (*GAL4*) to each experimental RNAi. * = *p* < 0.05, *** = *p* < 0.001, **** = *p* < 0.0001. ns = not significant. The graphs were plotted using GraphPad Prism. Scale bars = 1 s (horizontal), 50 μm (vertical) in **(A)** for all images.

While examining our intravital imaging, we noticed that the *mtRNase P* knockdown animal kymographs had an irregular appearance (Figure 7A, Supplementary Figure S12). Mitochondrial cardiomyopathy often manifests in arrhythmias. To quantify this and determine whether the reduction of mtRNase P caused heart rhythm abnormalities in addition to weak contractility, we examined heart rate (HR), heart period (HP), and calculated arrhythmicity indices (Supplementary Figure S11). *mtRNase P* RNAi using *Hand* GAL4 caused significantly decreased heart rate in males, whereas in females, only reduction of *rswl* showed any effect (Figure 7B). The heart period was increased in both females and males with a reduction of *rswl*, *scu,* and *mldr*, with *rswl* showing the largest effect (Figure 7C). We also calculated arrhythmicity indices, using heart period standard deviations (Figure 7D) (Ocorr et al., 2014). For both females and males, reduction of *rswl* and *mldr* caused significant increases in arrhythmicity, which can also be seen by examining the kymographs (Figure 7A, Supplementary Figure S11). At 2 days-old, *Hand* > *scu* ^*HMS*^ did not appear to have increased arrhythmicity but may acquire this phenotype with aging. Thus, reduction of each mtRNase P subunit affected HR and HP, but only *rswl* and *mldr* knockdown induced arrhythmia.

## Discussion

### *Drosophila* mtRNase P RNAi Knockdown as a Model for Mitochondrial Disease

In humans, point mutations in all three subunits of mtRNase P cause disease (Zschocke, 2012; Metodiev et al., 2016; Hochberg et al., 2021). In all cases, patient cells have reduced mt:tRNA processing, however, clinical symptoms are pleiotropic. Variable clinical presentation is true for many mitochondrial diseases which presents a challenge for physicians with respect to diagnoses and treatment (Gorman et al., 2016). There are currently no cures for mitochondrial disease, only palliative options. Since different patient tissues, such as skeletal, cardiac, and smooth muscle and the nervous systems, are affected with different severity, developing model systems to study mitochondrial diseases could prove useful for understanding basic disease etiology as well as potentially identifying improved, more targeted treatment options.

mtRNase P is highly conserved and *Drosophila* has good orthologs for each subunit (Figure 1A, (Sen et al., 2016). Protein null alleles of *rswl*, *scu,* and *mldr* are lethal (Saoji et al., 2021). Like patient cells, *Drosophila* null alleles of *mtRNase P* have mt:tRNA junction processing defects (Saoji et al., 2021). In this study, we took advantage of *Drosophila* genetics to study the reduction of *rswl*, *scu*, and *mldr* in a precise tissue-specific manner. The fly genotypes we have used do not abolish protein but merely reduce the levels. This has allowed a detailed analysis of the skeletal and cardiac muscle defects that arise in flies experiencing deficits in mtRNase P.

### Rswl, Scu and Mldr Reduction in Skeletal Muscle Causes Decreased Muscle Volume and Decreased Muscle and Mitochondrial Function

*Drosophila* has been a useful model to understand muscle development and disease (Bothe and Baylies, 2016). *Drosophila* adult muscles have a similar structure compared to vertebrates in that each muscle is composed of multiple fibers which are made up of many myofibrils. *Mef2* GAL4 expression occurs during all the muscle development. Using *Mef2* GAL4, we show that the knockdown of each mtRNase P subunit affects myofibril organization and structure. In all genotypes, sarcomere size was reduced. Previous studies have shown that increasing and decreasing insulin signaling in muscle during development increases and decreases adult body size and structures, respectively (Demontis and Perrimon, 2009). For muscle-specific *mtRNase P* knockdown, while we observed smaller muscles, fly body size appeared to remain the same. This suggests a different mechanism of regulation between modulating insulin signaling in muscles and specifically perturbing mitochondrial function (discussed below). While pupae and adults had smaller muscles, myofibrils in pupae (*rswl* ^*GD*^ and *scu* ^*HMS*^ RNAi) and adults (*scu* ^*GL*^ and *mldr* ^*KK*^ RNAi) had some phenotypic differences. *rswl* ^*GD*^ and *scu* ^*HMS*^ RNAi caused smaller muscles, but actin labeling indicated they remained intact. *scu* ^*GL*^ and *mldr* ^*KK*^ RNAi caused myofibrillar actin abnormalities including frayed actin and abnormal actin accumulation in sarcomeres. One possible explanation for these differences is that the adults use their muscles more than pupae, adding physical stress that could cause tears. The muscle defects we observed caused functional defects since negative geotaxis was adversely affected. In adults, *Mef2* > *scu* ^*GL*^ and *mldr* ^*KK*^ flies had abnormal wing posture, but there was no indication of collapsed thoraces. This suggests that reduced mtRNase P function does not cause muscle degeneration (Deak, 1977).

In muscle, mitochondria form a complex network between myofibrils that is important for maintaining energy availability (Glancy et al., 2017, 2020). In *Drosophila*, myofibril morphogenesis is coordinated with mitochondrial network formation during muscle development (Avellaneda et al., 2021). This coordination is disrupted when mitochondrial fission/fusion is perturbed which alters mitochondrial intercalation, leading to impaired muscle development (Avellaneda et al., 2021). Reducing mtRNase P using *Mef2* GAL4 expression resulted in a concomitant reduction of thoracic ATP. In *rswl* ^*GD*^ and *scu* ^*HMS*^ RNAi pupae, mitochondria lost their stereotypical regular morphology and distribution. As *Mef2* expression occurs during muscle development, the myofibril disorganization and decreased sarcomere size we observed in pupae could be due to decreased mitochondrial output available during development that potentially does not allow mitochondrial intercalation. Mitochondrial morphology is different in *Mef2* > *scu* ^*GL*^ and *mldr* ^*KK*^ RNAi adults. The organelles become rounder and less compact between the myofibrils. With the loss of negative geotaxis and reduced ATP, these adults clearly have reduced mitochondrial function that impacts muscle function. In addition to reduced energy, the decreased mitochondrial function could impact myofibril structure due to decreased maintenance or failure to initiate mitochondrial intercalation (Avellaneda et al., 2021).

### Rswl and Mldr Reduction in the *Drosophila* Heart Causes Heart Dilation and Arrhythmia

Since cardiac muscles demand high energy like skeletal muscle, mitochondrial diseases are often associated with cardiomyopathy. Children and adults with mitochondrial disease experience cardiomyopathy, with a particularly high incidence (20%–40%) in children (El-Hattab and Scaglia, 2016). The most prevalent symptoms are hypertrophic cardiomyopathy; however, dilated cardiomyopathy and arrhythmias are also frequently seen (Duran et al., 2019). *Drosophila* and human heart development share conserved regulatory networks and *Drosophila* has been a useful model to study heart defects associated with disease (Taghli-Lamallem et al., 2016; Petersen et al., 2020).

Knockdown of each mtRNase P subunit using heart-specific *Hand* GAL4 did not affect development. We did observe significantly reduced lifespan as well as adult wing defects for *Hand* > *rswl* ^*GD*^, *scu* ^*HMS*^, *scu* ^*GL*^_*,*_ and *mldr* ^*KK*^ flies, indicating expressing the RNAi constructs resulted in phenotypic effects in heart-derived tissues. Heart myofibril structure was also greatly disrupted, and mitochondria were larger and more swollen after 1 week. While mtRNase P knockdown in skeletal muscle caused similar muscle defects, knockdown in the heart revealed subunit-specific effects. Using intravital imaging, we found a reduction of *rswl*, *scu,* and *mldr* caused decreased heart contractility. In contrast, only reduction of *rswl* and *mldr* resulted in arrhythmicity. These deficits recapitulate the symptoms seen in patients suffering from mitochondrial diseases, as well as the two cases of children with MRPP1 (Rswl) mutations and MRPP2 (Scu) (Zschocke, 2012; Metodiev et al., 2016). We do not yet know whether mtRNase P loss causes cardiac hypertrophy (Petersen et al., 2022).

### Differential Effects on Mitochondrial Function With Reduction of Individual mtRNase P Subunits

Since mtRNase P functions as a three-protein complex, the expectation might be that loss of any subunit would cause complete loss of function and thus the same phenotype. The results here suggest that there are tissue-specific differential effects *in vivo* for loss of mtRNase P subunits. This would align with the clinical manifestations in people. We found a similar subunit differential result for mt:tRNA junction processing. Null alleles for all three subunits differentially affect junction processing in different mtRNA contexts (Saoji et al., 2021). It is not clear why these differential effects take place *in vivo*, or how this mechanism would work. One possibility is that there are tissue-specific cofactors that aid in mitochondrial transcript binding and cleavage to improve cleavage efficiency. Evidence for cofactors has recently been shown *in vitro* (Karasik et al., 2021). As for Rswl, work in cell culture supports that loss of MRPP1 was most detrimental for mt:tRNA processing (Sanchez et al., 2011), with the supposition being that MRPP1-derived methylation could be the reason. The MRPP1/2 subcomplex is required for methylation and loss of MRPP2 results in decreased MRPP1 (Holzmann et al., 2008; Vilardo et al., 2012; Deutschmann et al., 2014). However, the evidence so far supports that methylation is not necessary for mt:tRNA cleavage (Vilardo et al., 2012). The structure of human mtRNase P bound to pre-mt:tRNA has recently been solved to 3.0 Å (Bhatta et al., 2021). The structure indicates human pre-mt:tRNA^Tyr^ associates with the MRPP2 tetramer, and one copy of MRPP1 and 3. It shows the enzymatic sites of MRPP2 and MRPP3 simultaneously contact their respective substrates thus methylation is not necessarily required for MRPP3 to contact the mt:tRNA nucleotides that will be cleaved. Future studies specifically examining the timing of methylation *in vivo* are required, as well enzymatic assays using an extract from different tissues to identify differences in processing and potential cofactors.

### Using Tissue-Specific Knockdown on mtRNase P in *Drosophila* to Better Understand Disease

Mitochondrial diseases are devastating, with only palliative care options available. Since RNAi knockdown of mtRNase P subunits in *Drosophila* recapitulates many of the tissue-specific disease symptoms, this model offers the opportunity to better understand mtRNase P function *in vivo*. *Drosophila* has been used to model mitochondrial diseases and heart function separately, but how mitochondrial dysfunction affects cardiac function in flies has not been as well explored. Reducing the amount of Rswl, Scu, and Mldr separately in skeletal and cardiac muscle offers the opportunity to test how this affects mt:tRNA junction cleavage at different developmental time points, as well as probe how reducing one protein, affects the stoichiometry of the complex. Future studies could screen small compounds to increase mt:tRNA cleavage rates that could stabilize or increase mt:tRNA binding *in vivo*, as well as create pathological patient point mutations in Rswl, Scu, and Mldr to better understand their effect *in vivo*.

## Data Availability

The original contributions presented in the study are included in the article/Supplementary Materials, further inquiries can be directed to the corresponding author.

## Abbreviations

mtRNAse P, Mitochondrial RNase P; mt:tRNA, mitochondrial tRNA; MRPP, Mitochondrial RNase P Protein; Rswl, Roswell; Scu, Scully; Mldr, Mulder; DLM, Dorsal Longitudinal Muscles; EDD, end-diastolic dimension; ESD, end-systolic dimension.
