# Supplementary figures and images for: Reduction of Drosophila Mitochondrial RNase P in Skeletal and Heart Muscle Causes Muscle Degeneration, Cardiomyopathy, and Heart Arrhythmia
Source: Front Cell Dev Biol. 2022 May 19;10:788516. doi: 10.3389/fcell.2022.788516 (PMC9162060; doi:10.3389/fcell.2022.788516)

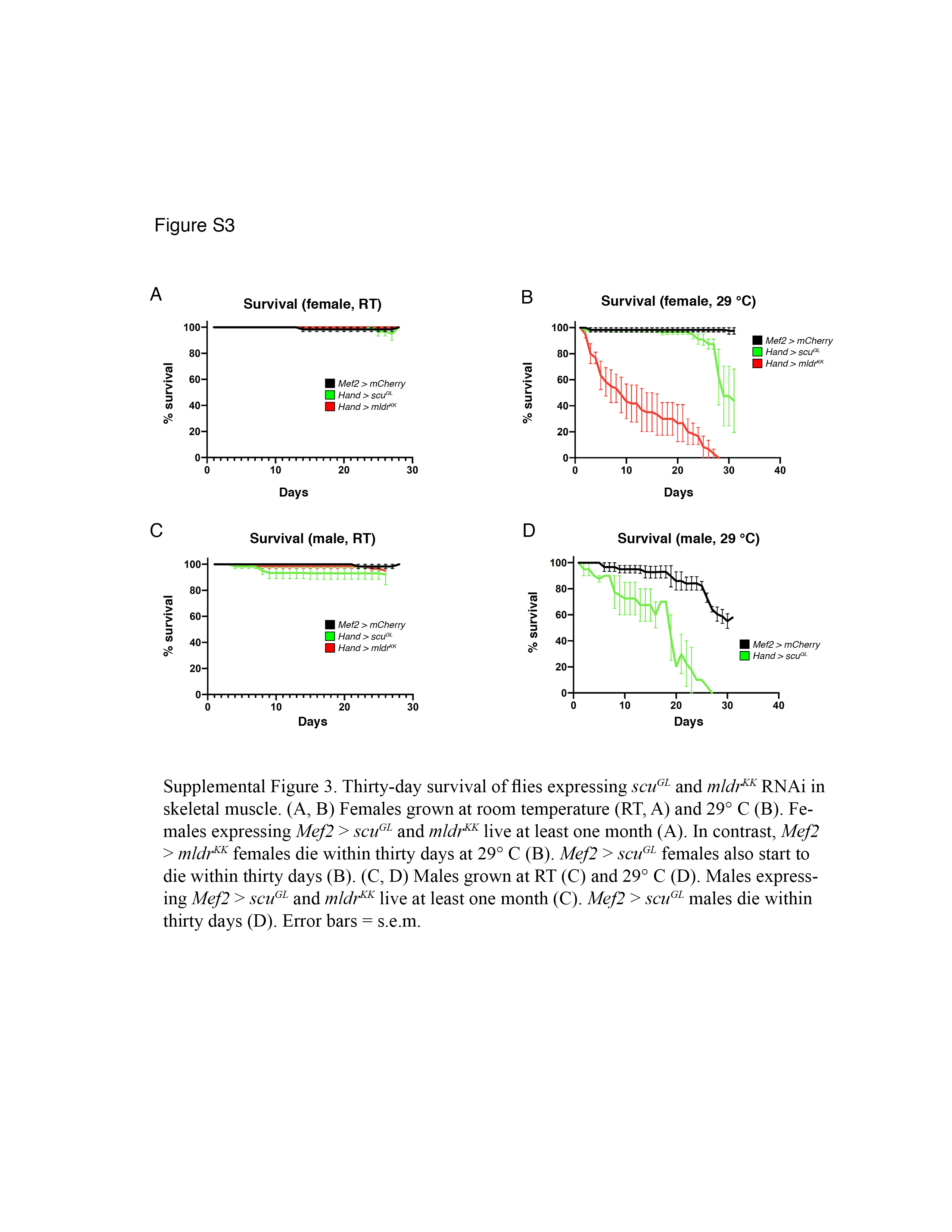

Supplement: Supplementary file 1 [file Image3.JPEG]

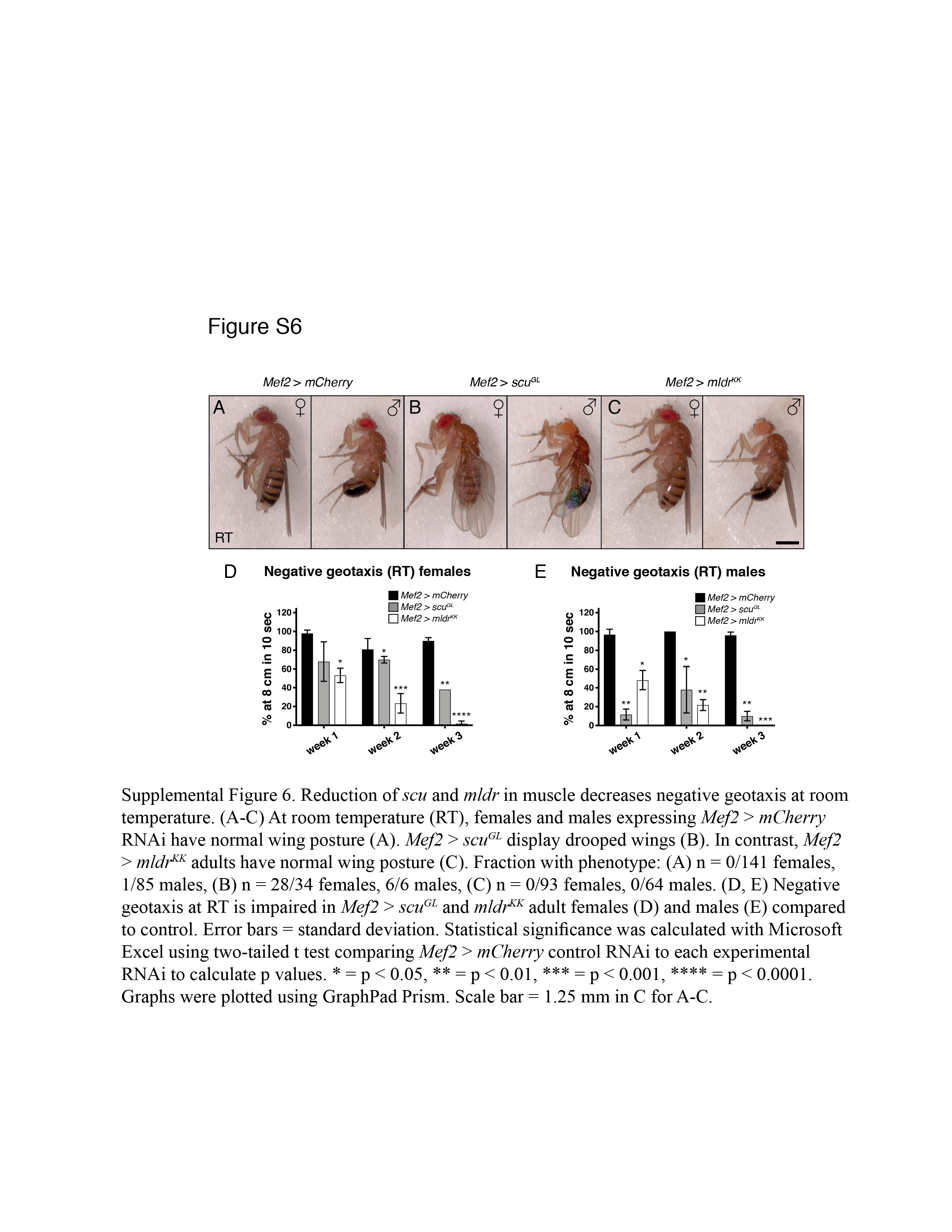

Supplement: Supplementary file 2 [file Image6.jpg]

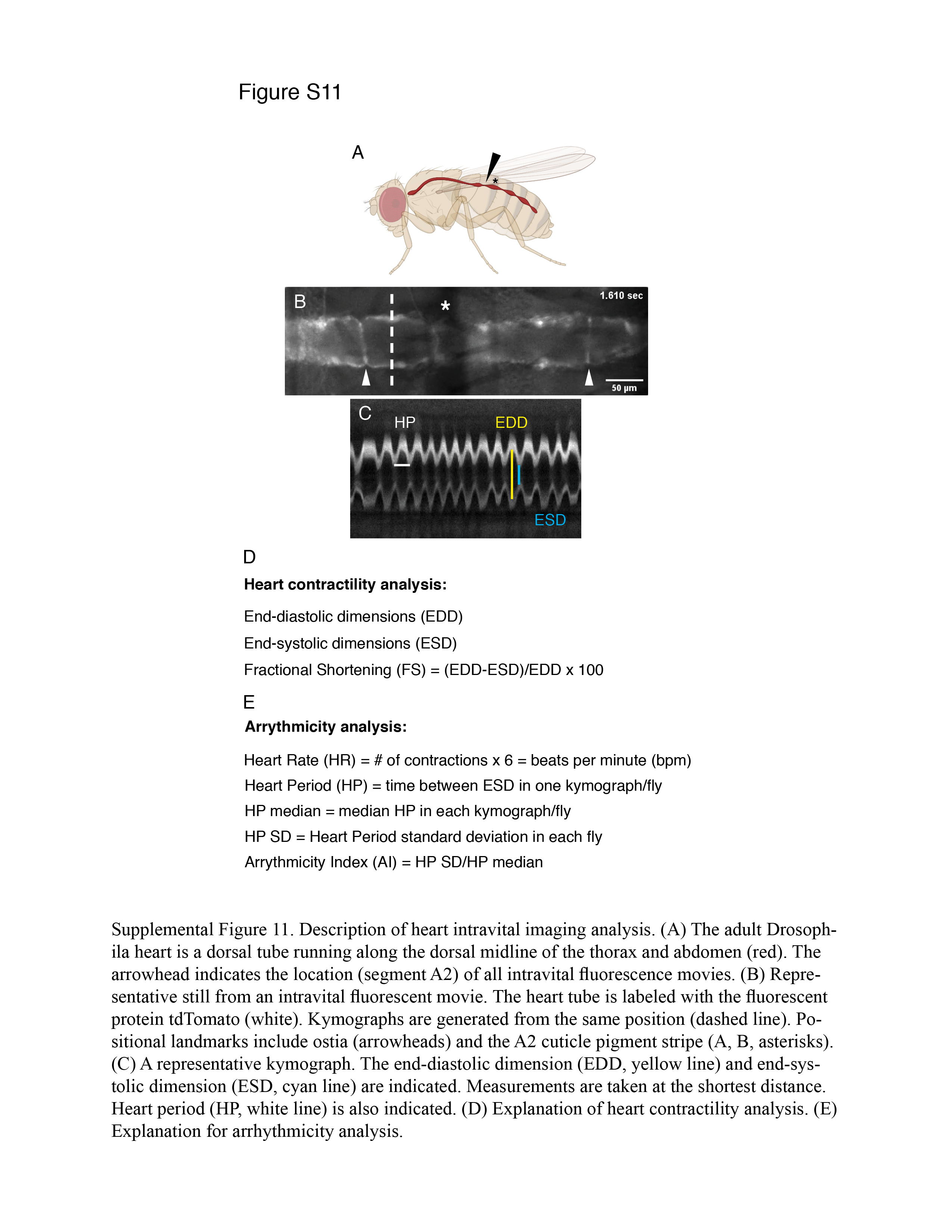

Supplement: Supplementary file 3 [file Image11.jpg]

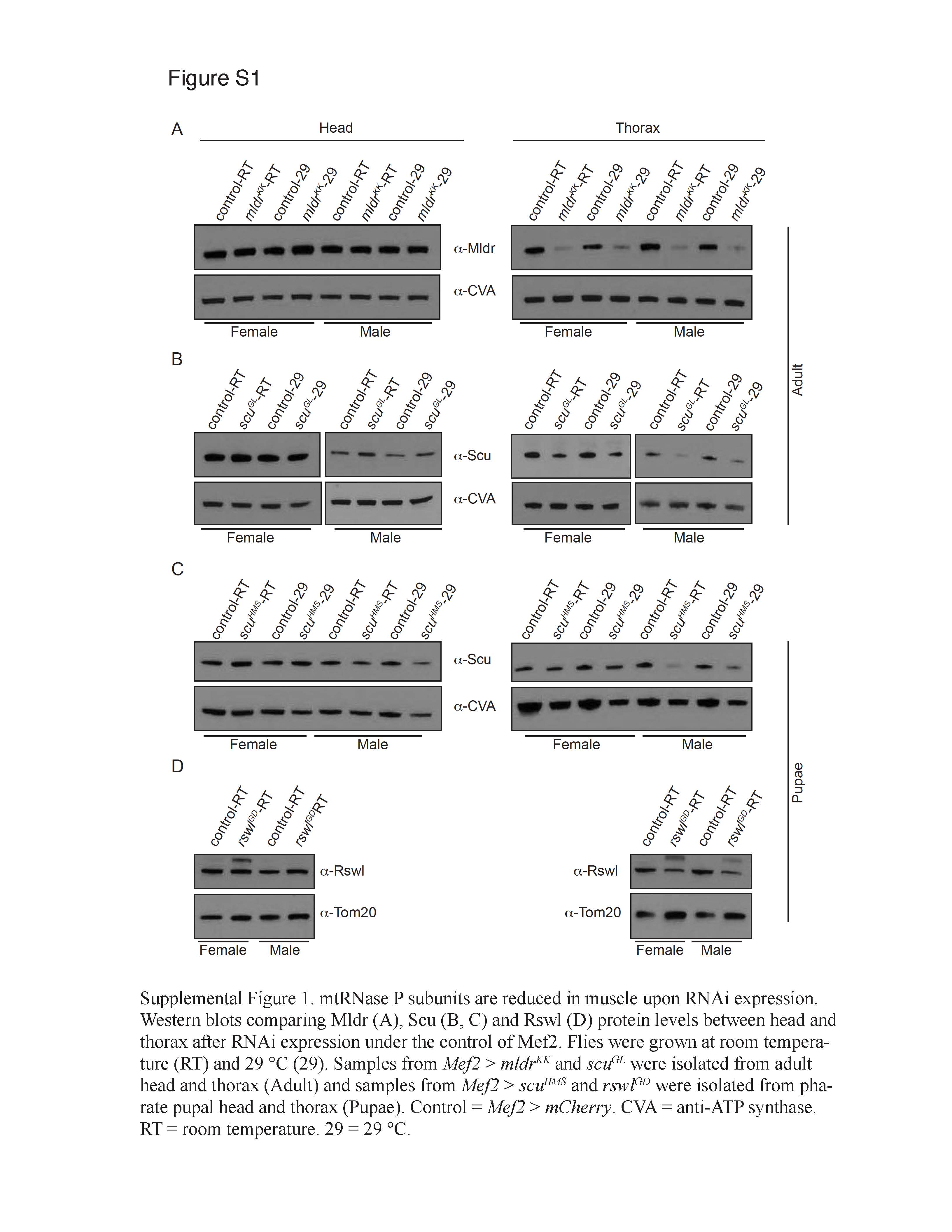

Supplement: Supplementary file 4 [file Image1.JPEG]

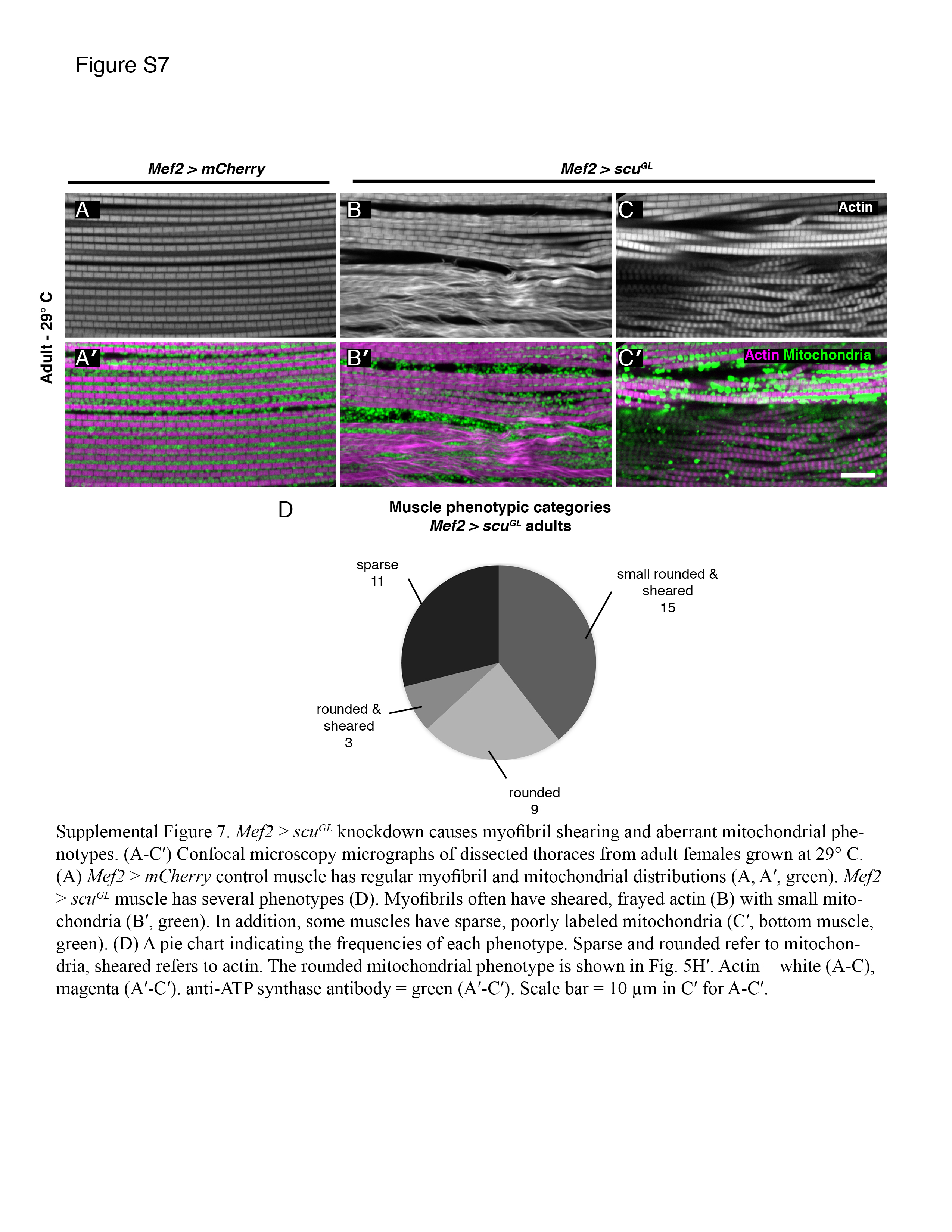

Supplement: Supplementary file 5 [file Image7.JPEG]

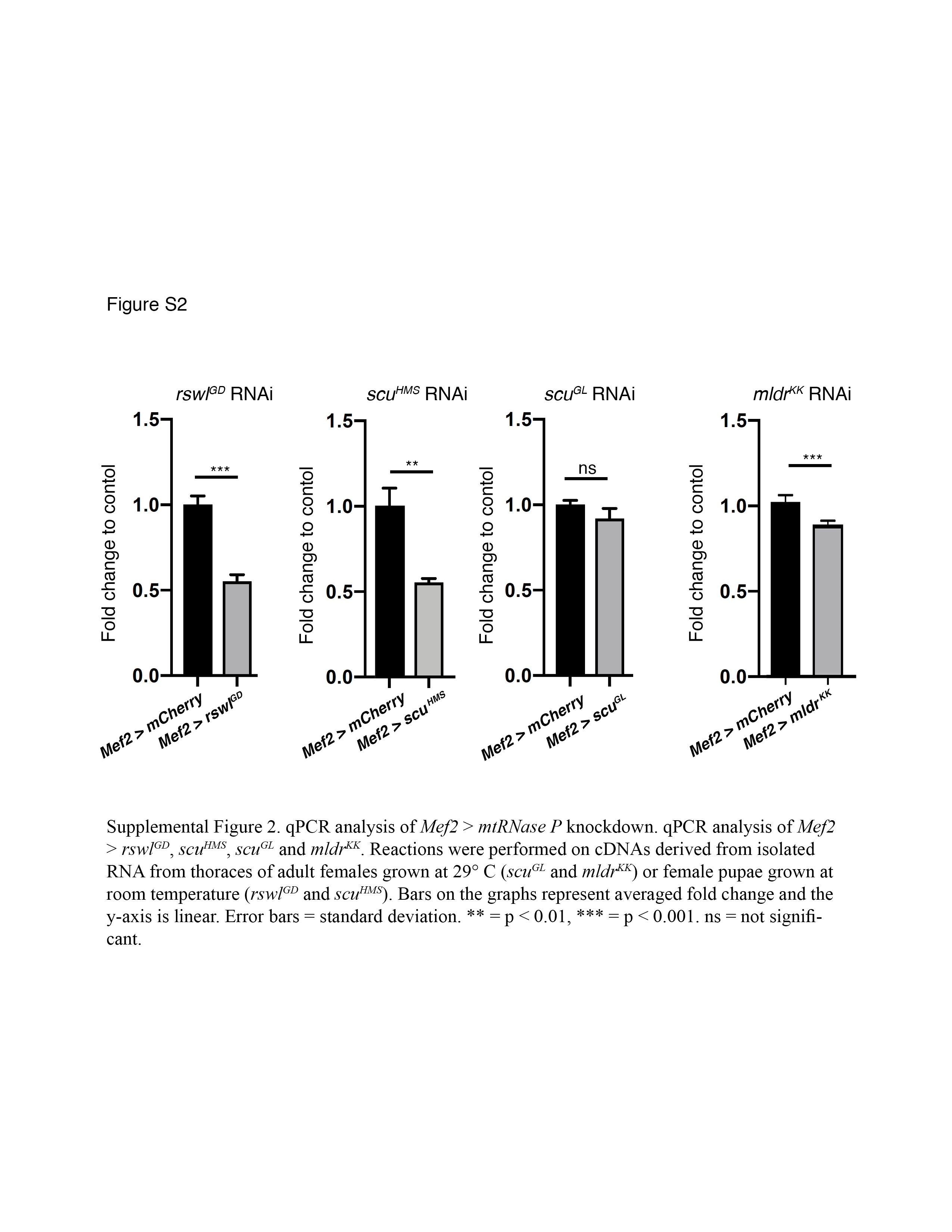

Supplement: Supplementary file 6 [file Image2.JPEG]

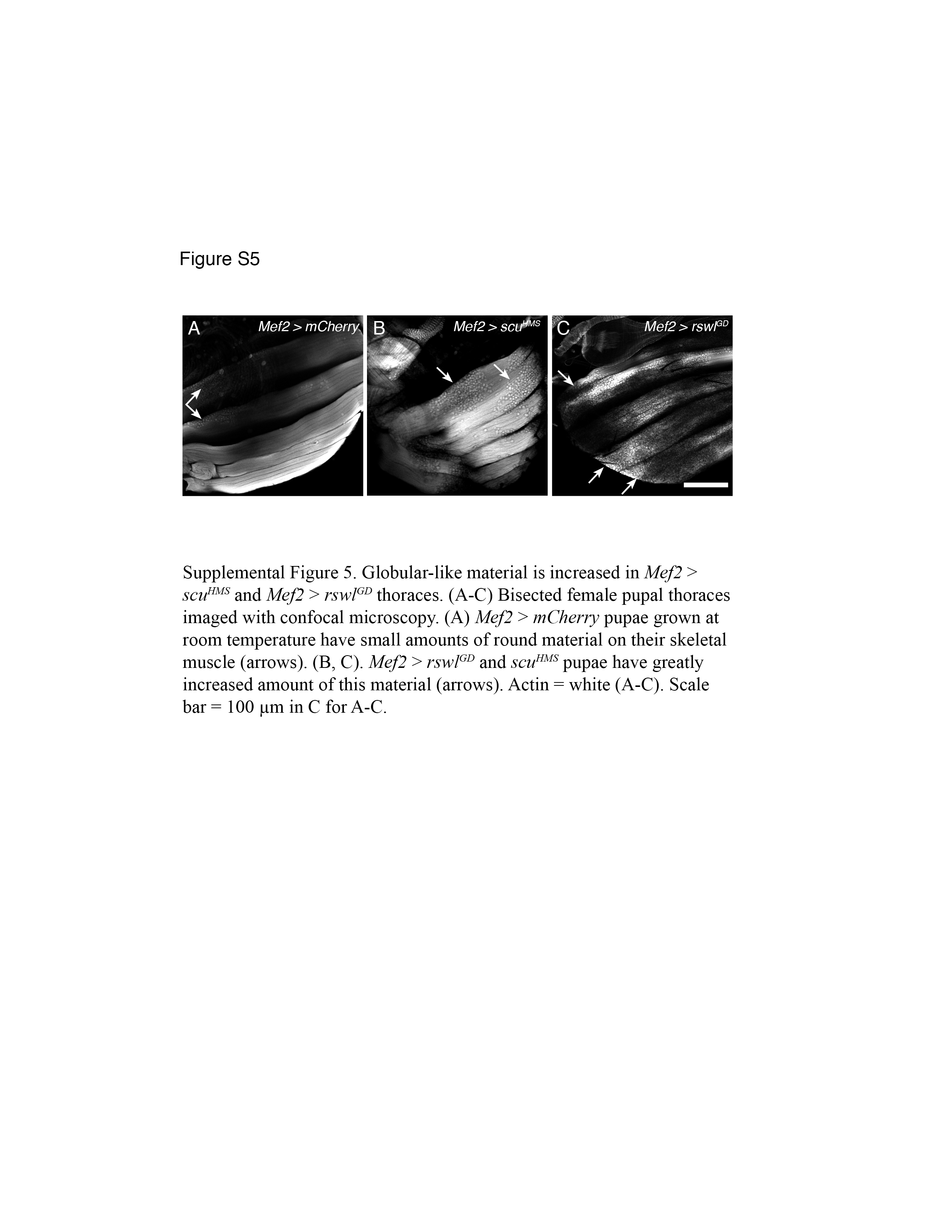

Supplement: Supplementary file 7 [file Image5.JPEG]

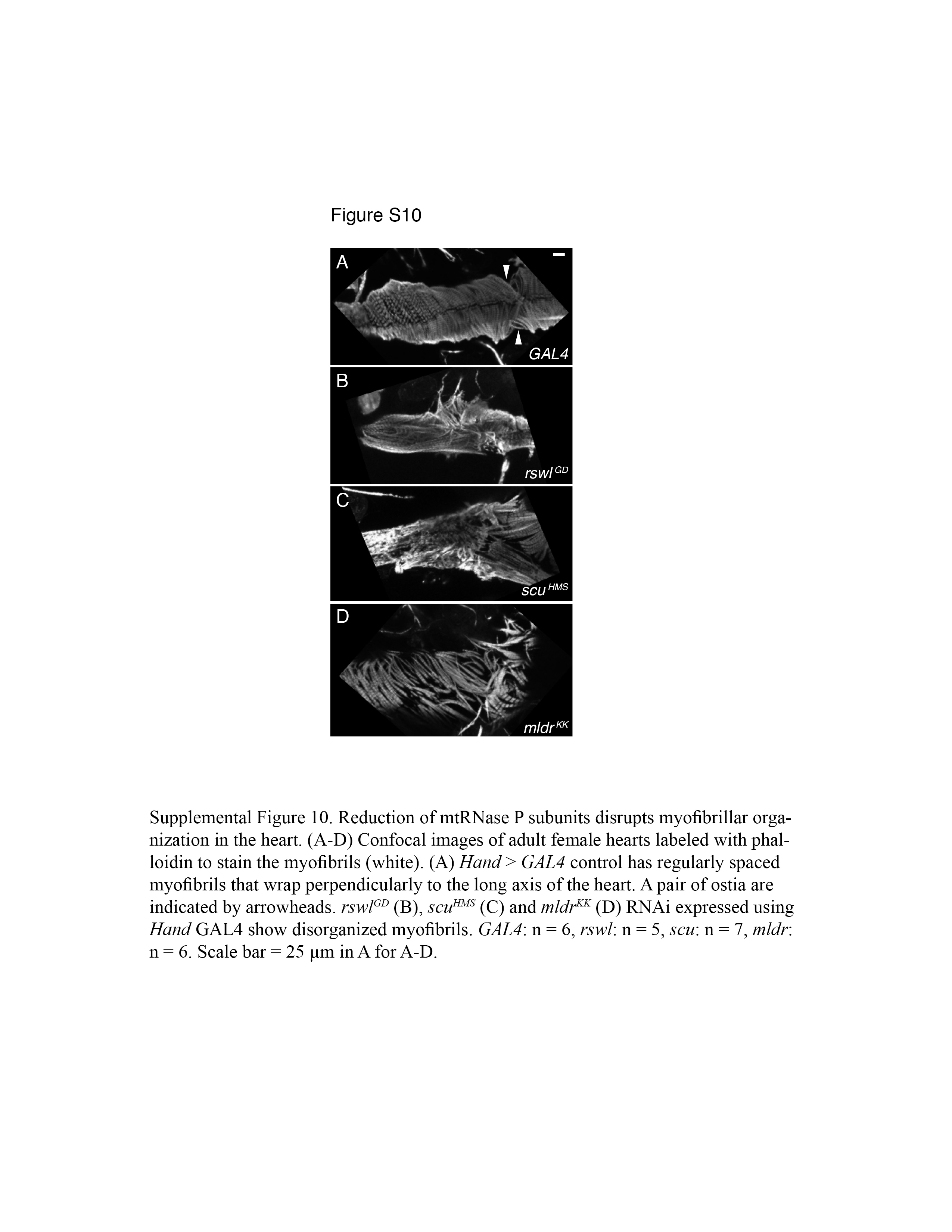

Supplement: Supplementary file 8 [file Image10.JPEG]

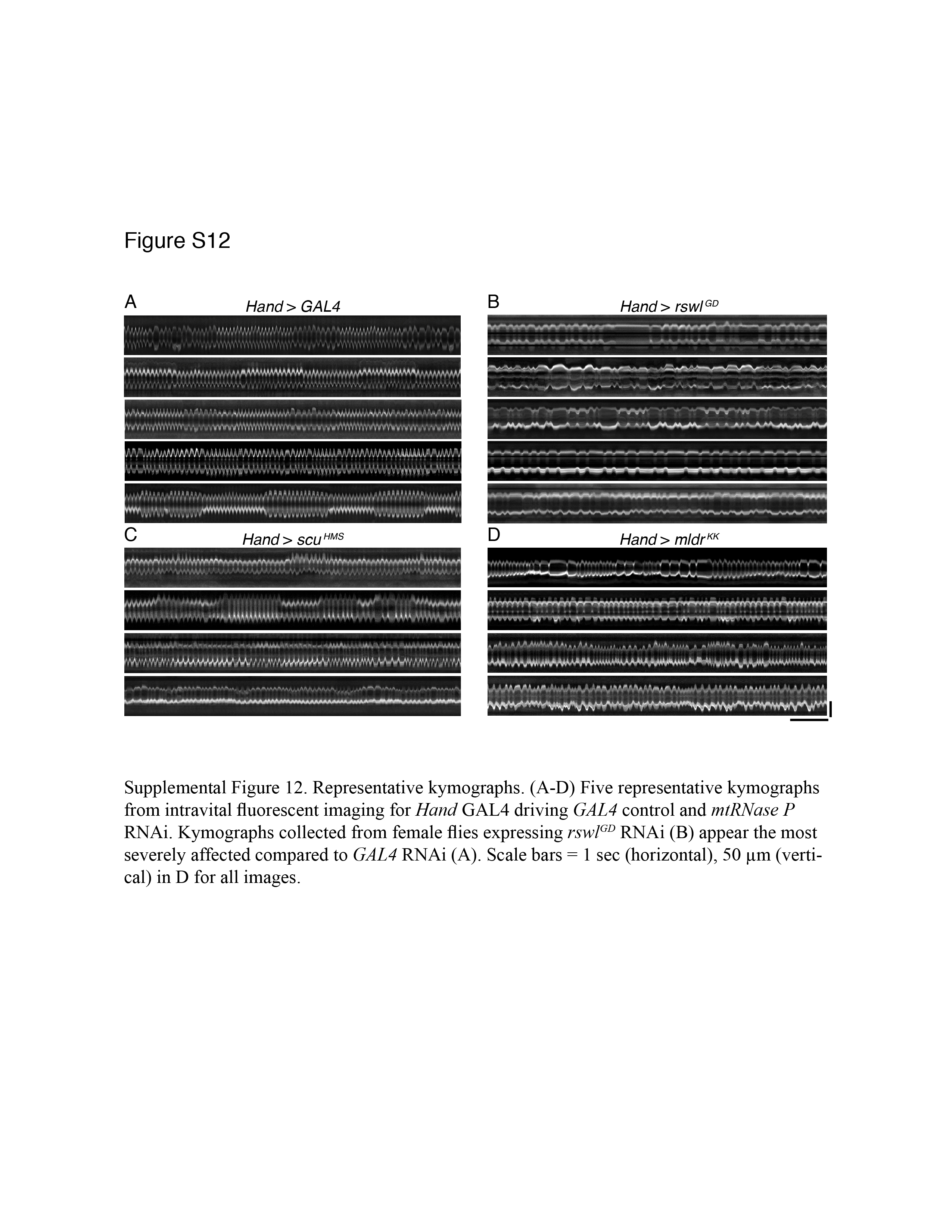

Supplement: Supplementary file 9 [file Image12.JPEG]

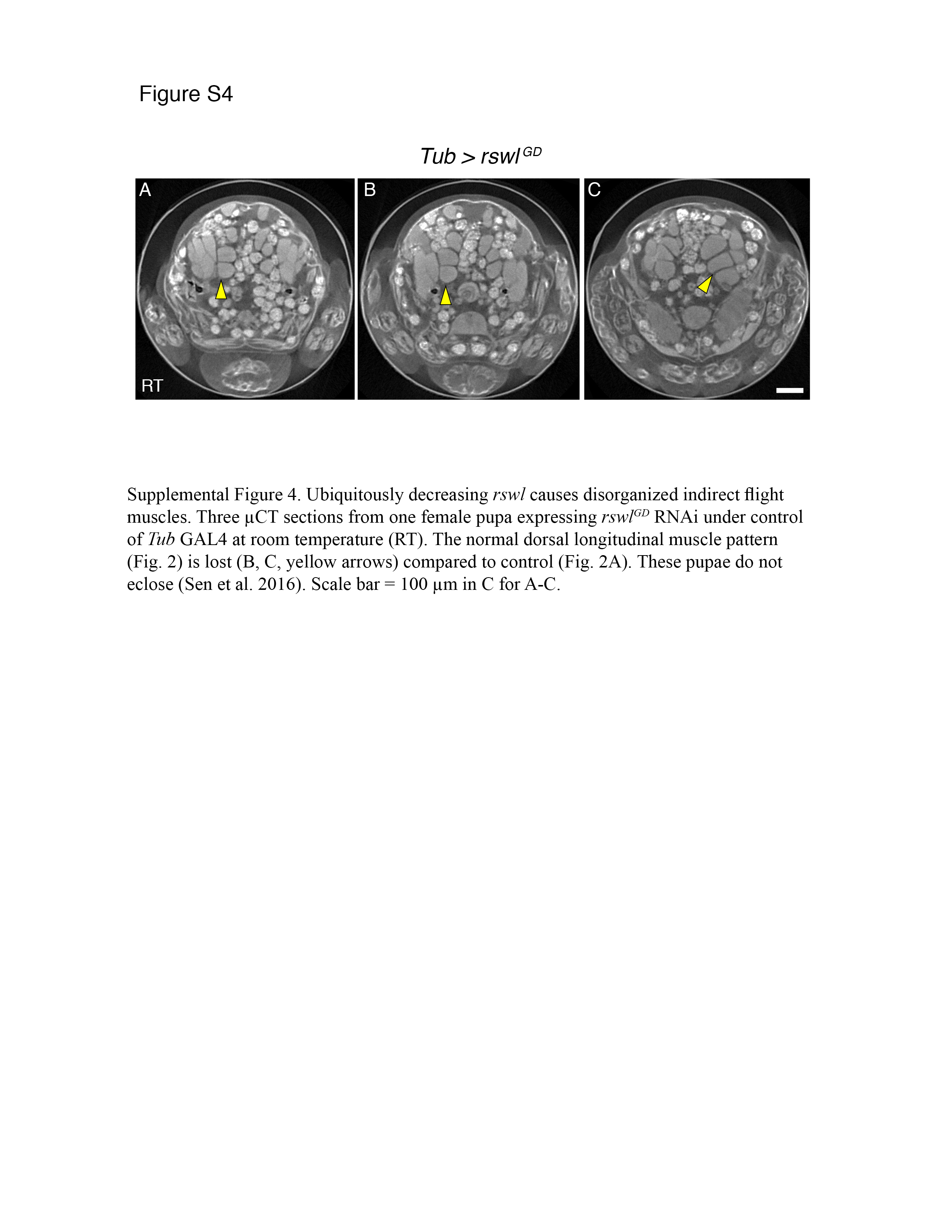

Supplement: Supplementary file 10 [file Image4.jpg]

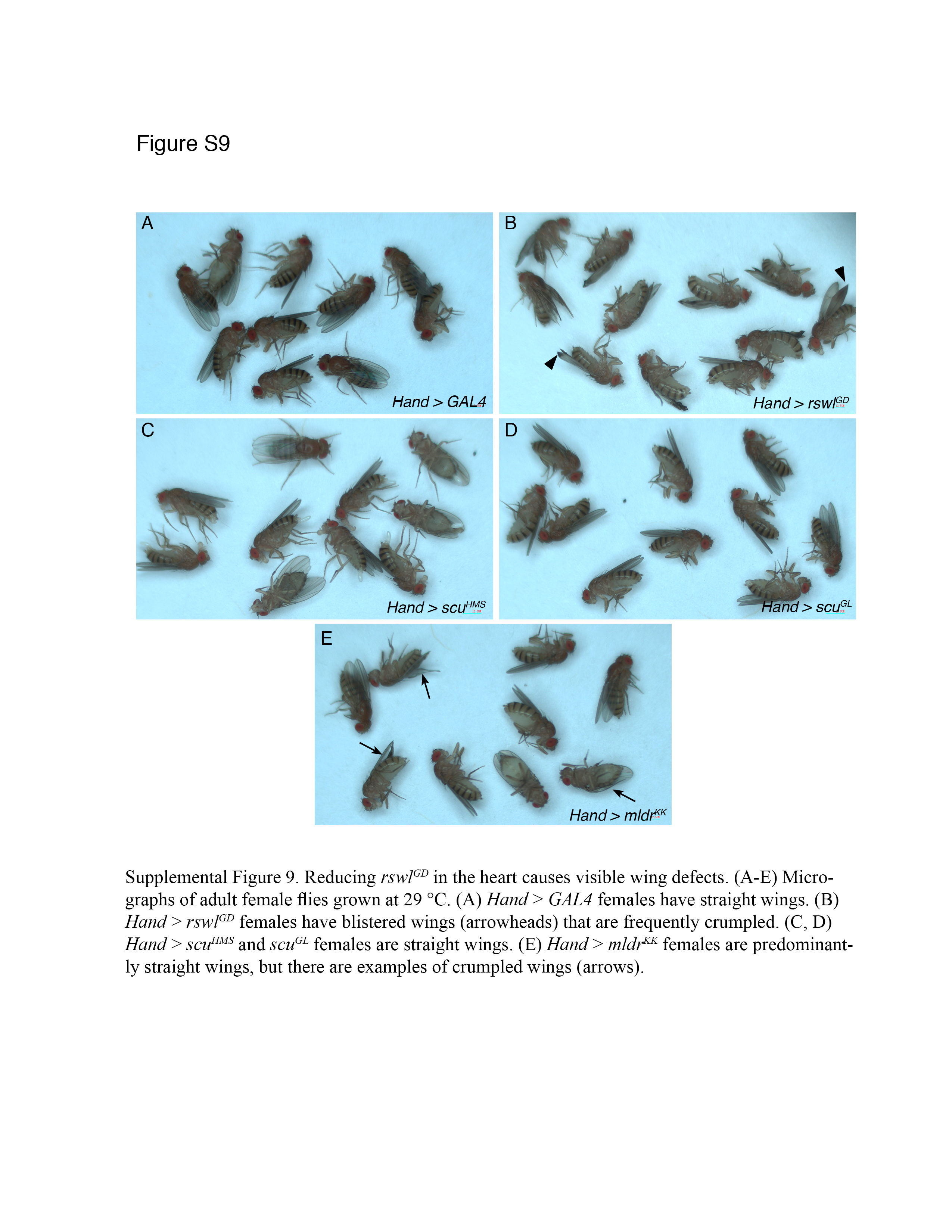

Supplement: Supplementary file 11 [file Image9.jpg]

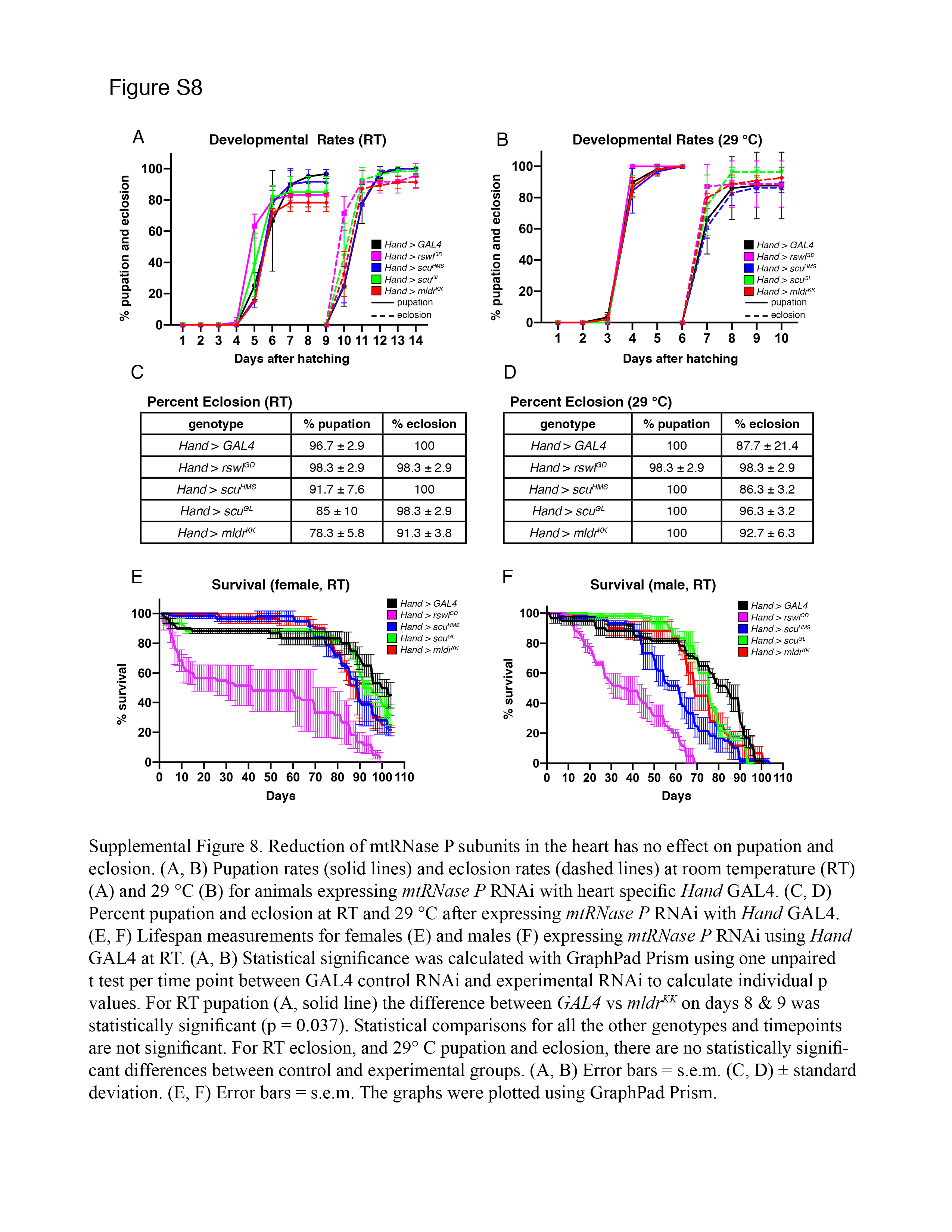

Supplement: Supplementary file 12 [file Image8.jpg]
